# Synthesis, molecular docking and biological potentials of new 2-(4-(2-chloroacetyl) piperazin-1-yl)-*N*-(2-(4-chlorophenyl)-4-oxoquinazolin-3(4*H*)-yl)acetamide derivatives

**DOI:** 10.1186/s13065-019-0629-0

**Published:** 2019-09-05

**Authors:** Shinky Mehta, Sanjiv Kumar, Rakesh Kumar Marwaha, Balasubramanian Narasimhan, Kalavathy Ramasamy, Siong Meng Lim, Syed Adnan Ali Shah, Vasudevan Mani

**Affiliations:** 1 Faculty of Pharmaceutical Sciences, Maharshi Dayanand University, Rohtak, 124001 India; 2 Faculty of Pharmacy, Universiti Teknologi MARA (UiTM), 42300 Bandar Puncak Alam, Selangor Darul Ehsan Malaysia; 3 Collaborative Drug Discovery Research (CDDR) Group, Pharmaceutical Life Sciences Community of Research, Universiti Teknologi MARA (UiTM), 40450 Shah Alam, Selangor Darul Ehsan Malaysia; 4 Atta-ur-Rahman Institute for Natural Products Discovery (AuRIns), Universiti Teknologi MARA, 42300 Bandar Puncak Alam, Selangor Darul Ehsan Malaysia; 5 Department of Pharmacology and Toxicology, College of Pharmacy, Qassim University, Buraidah, 51452 Kingdom of Saudi Arabia

**Keywords:** Quinazolinones, Antimicrobial, Anticancer potential, HCT116, RAW264.7, Molecular docking

## Abstract

In the present study, a series of 2-(4-(2-chloroacetyl)piperazin-1-yl)-*N*-(2-(4-chlorophenyl)-4-oxoquinazolin-3(4*H*)-yl)acetamide derivatives was synthesized and its chemical structures were confirmed by physicochemical and spectral characteristics. The synthesized compounds were evaluated for their in vitro antimicrobial (tube dilution technique) and anticancer (MTT assay) activities along with molecular docking study by Schrodinger 2018-1, maestro *v11.5*. The antimicrobial results indicated that compounds **3**, **8**, **11** and **12** displayed the significant antimicrobial activity and comparable to the standards drugs (ciprofloxacin and fluconazole). The anticancer activity results indicated that compound **5** have good anticancer activity among the synthesized compounds but lower active than the standard drugs (5-fluorouracil and tomudex). Molecular docking study demonstrated that compounds **5** and **7** displayed the good docking score with better anticancer potency within the binding pocket and these compounds may be used as a lead for rational drug designing for the anticancer molecules.
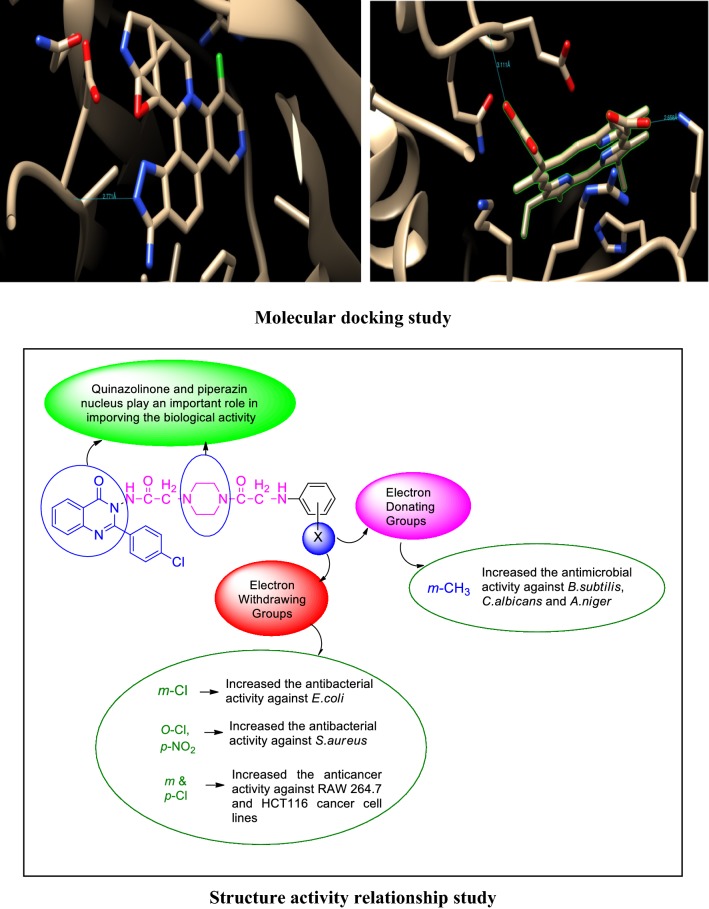

## Introduction

The ever-increasing microbial antibiotic resistance leads to ongoing testing of new biologically efficient compounds of either natural or synthetic origin for infectious diseases [1]. Subsequently, compounds carrying heterocyclic nuclei gained much attention in the growth of novel antimicrobial agents due to their chemotherapeutic significance. Quinazoline nucleus is an exciting molecule with two nitrogen atoms in its structure among the most significant classes of aromatic bicyclic compounds. Quinazoline is one of the most widespread scaffolds among natural and synthetic bioactive compounds. Quinazoline heterocyclic compound resembles both the purine nucleus and the pteridine one and exhibited wide spectrum medicinal values i.e. antihypertensive, antitumor, antiplasmodial, antiviral and anti-inflammatory activities [2, 3].

**Fig. 1:**
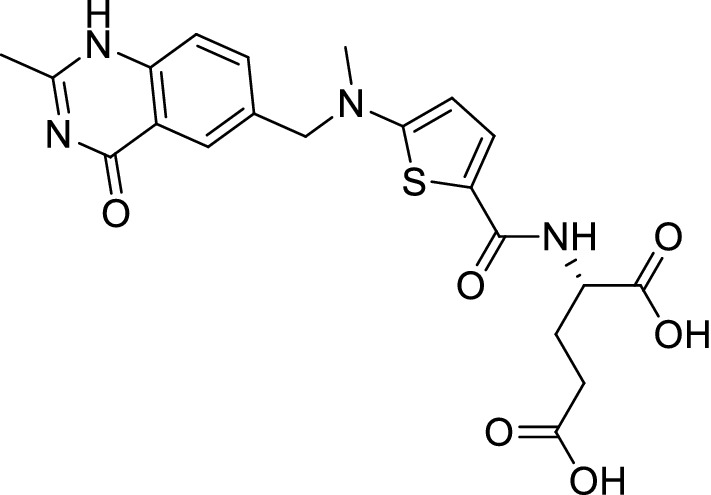
Structure of Raltitrexed (Tomudex)

Interest in quinazolinones as anticancer agents has further increased since the discovery of raltitrexed (Tomudex^®^) (Fig. 1) as an antimetabolite drug used in cancer chemotherapy. Quinazolinone derivatives have been reported to have potent anticancer activities viz. aurora kinase inhibitors [4], α-folate receptor inhibitors [5], CDK-inhibitors [6], activin-like kinase (ALK) inhibitors [7], EGFR inhibitors [8], topoisomerase inhibitors [9], pin1 (Protein interaction with NIMA1) inhibitors [10], T cell proliferation inhibitors on peripheral blood mononuclear cells (PBMC) and jurkat cells [11] and VEGFR inhibitors [12].

Molecular docking analyses provide the most comprehensive illustration of the interaction between drug receptors and produced a modern rational approach to drug design [13]. The RAW 264.7 cells are monocyte/macrophage like cells from the BALB/c mouse modified cell line from Abelson leukemia virus. These cells are defined as a suitable macrophage model. Pinocytosis and phagocytosis can be performed. RAW 264.7 cells raise the production of nitric oxide (NO) at LPS stimulation and improve phagocytosis. In addition, these cells can destroy target cells by cytotoxicity dependent on antibodies [14]. Macrophages are immune cells found in many distinct tissues, performing a broad variety of biological activities. They are highly plastic in their pattern of protein expression and can be activated by a variety of cytokines and pathogen-associated molecules like lipopolysaccharide. The quinazoline and other heterocyclic compounds reported have a mass variety of less than 500 ppm or more [15–18].

Recently Kiruthiga et al. [19] reported that quinazolinone moiety is (**I**) essential for antimicrobial activity. Rajveer et al. [20] found that acetamide nucleus attached at *N*-1 position to quinazoline moiety (**II**) possessed good antimicrobial activity. Rajasekaran et al. [1] proposed that acetamide group of quinazolinone moiety (**III**) attached with heterocyclic compound showed better antimicrobial activity. Kumar et al. [21] found that halogenated phenyl moiety (chlorine substitution at the 2nd position) (**IV**) attached at 2nd position on the quinazolinone nucleus possesses a good antimicrobial activity. Desai et al. [3] showed that quinazolinones bearing piperazine moiety (**V**) having asserted antimicrobial activity.

**Fig. 2:**
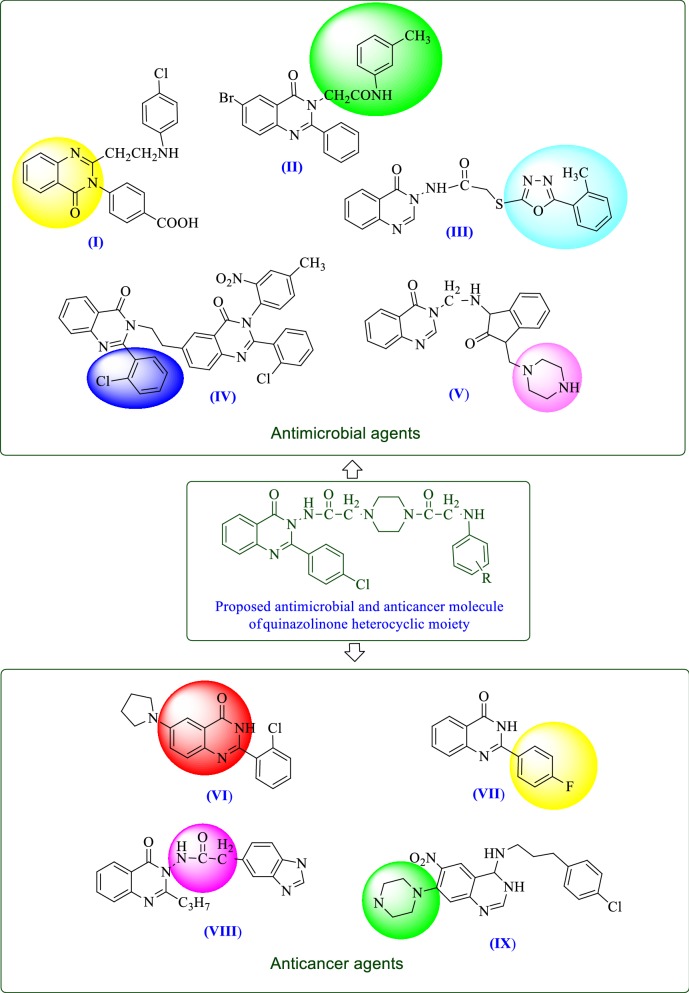
Design of proposed quinazolinone molecules based on literature

Hour et al. [22] identified that the presence of phenyl ring at 2nd position of quinazolinone nucleus (**VI**) improved the anticancer activity. Xia et al. [23] revealed that substitution with halogens at 4th position on phenyl ring attached at 2nd position of quinazolinone moiety (**VII**) increased anticancer activity. Raghavendra et al. [24] reported that acetamide group at *N*-1 position of quinazolinone nucleus (**VIII**) enhanced the anticancer activity. Tobe et al. [25] reported that quinazolinone nucleus having a piperazine ring substitution (**IX**) exerted an anticancer activity by suppression of T cell proliferation (Fig. 2).

### Rational behind the selection of cyclin dependent kinase (CDK8)

Quinazolin derivatives are protein kinase inhibitors. Protein kinases are the most important class of human enzymes that regulate the sequence of events such as cell cycle progression, cell division and cell proliferation [26]. Developing new quinazoline derivatives as an anticancer agent is considered a promising area and researchers around the world are continuously exploring this region to generate new drug candidates [26]. CDK activity is controlled by association with CDK-inhibiting regulatory subunits (cyclines) and proteins, their phosphorylation status and ubiquitin-mediated proteolysis. Since the loss of cell cycle control leading to deregulated cell proliferation is one of cancer’s hallmarks, it is anticipated that the inhibition of CDKs will provide an effective tumor growth control strategy and thus impact cancer therapy. Many organisations researched CDK inhibition and used a range of structural templates with different degrees of selectivity and activity [27, 28].

In the light of above facts and in continuation of our effort to develop novel anticancer and antimicrobial agents [29–31], the present study is aimed to design, molecular docking and synthesize of 2-(4-(2-chloroacetyl)piperazin-1-yl)-*N*-(2-(4-chlorophenyl)-4-oxoquinazolin-3(4*H*)-yl)acetamides as prospective anticancer and antimicrobial agents.

## Experimental

### Materials and methods

Starting materials were obtained from commercial sources and used without further purification. The microbial strains for the antimicrobial evaluation were obtained from the Microbial Type Culture Collection and Gene Bank MTCC, Chandigarh. Thin layer chromatography (TLC) using commercial silica gel plates (Merck), Silica gel F254 on aluminum sheets, has noted reaction improvements. Infrared (KBr pellets, cm^−1^) spectra were recorded on an Agilent Resolutions Pro FT-IR spectrometer. Melting points were determined in open capillary tubes. Mass spectra were recorded using Waters Micromass Q-Tof micro instrument. ^1^H-NMR (DMSO) and ^13^C–NMR (DMSO) were recorded at 600 MHz and 150 MHz, respectively on Bruker Avance III 600 NMR spectrometer. Perkin–Elmer 2400 C, H and N analyzer used for elemental analysis.

### Synthetic procedure for the synthesis of quinazolinone derivatives (1–17)

#### *2*-*(4*-*Chlorophenyl)*-*4H*-*benzo[e] [*1, 3*] oxazin*-*4*-*one* (**I**)

2-Aminobenzoic acid (0.01 mol) was stirred for 3 h at room temperature with 4-chloro benzoyl chloride (0.01 mol) in the presence of pyridine. The resultant mixture was treated with 5% sodium bicarbonate solution to get **I**, which was filtered, dried and recrystallized with ethanol.

#### *3*-*Amino*-*2*-*(4*-*chlorophenyl)quinazolin*-*4(3H)*-*one* (**II**)

2-(4-Chlorophenyl)-4*H*-benzo[e] [1, 3] oxazin-4-one (**I**) (0.01 mol) was reacted with hydrazine hydrate (0.02 mol) in the presence of ethanol and refluxed for 3 h (30 °C) to obtain **II**. The reaction mixture was cooled and the resultant precipitate was filtered off and recrystallized with ethanol [32].

#### *2*-*Chloro*-*N*-*(2*-*(4*-*chlorophenyl)*-*4*-*oxoquinazolin*-*3(4H)*-*yl)acetamide* (**III**)

A mixture of 3-amino-2-(4-chlorophenyl)quinazolin-4(3*H*)-one (**II**) (0.01 mol) and chloroacetyl chloride (0.01 mol) with a few drops of glacial acetic acid in absolute ethanol (20 mL) was refluxed for 8 h (30 °C). The reaction mixture was then cooled in ice cold water and resultant precipitate of **III** was filtered, washed with water, dried and recrystallized with ethanol.

#### *N*-*(2*-*(4*-*Chlorophenyl)*-*4*-*oxoquinazolin*-*3(4H)*-*yl)*-*2*-*(piperazin*-*1*-*yl)acetamide* (**IV**)

A mixture of 2-chloro-*N*-(2-(4-chlorophenyl)-4-oxoquinazolin-3(4*H*)-yl)acetamide (**III**) (0.01 mol) and piperazine (0.01 mol) with a few drops of glacial acetic acid in absolute ethanol (20 mL) was refluxed for 10 h (40 °C). The reaction mixture was cooled in ice cold water and resultant precipitate of **IV** was filtered, washed with water, dried and recrystallized with ethanol.

#### *2*-*(4*-*(2*-*Chloroacetyl)piperazin*-*1*-*yl)*-*N*-*(2*-*(4*-*chlorophenyl)*-*4*-*oxoquinazolin*-*3(4H)*-*l) acetamide* (**V**)

*N*-(2-(4-Chlorophenyl)-4-oxoquinazolin-3(4*H*)-yl)-2-(piperazin-1-yl)acetamide (**IV**) (0.01 mol) was further treated with chloroacetyl chloride (0.01 mol) in the presence of few drops of glacial acetic acid in absolute ethanol (20 mL) and refluxed for 8 h (30 °C) yielded of **V**. The reaction mixture was then cooled in ice cold water and resultant precipitate was filtered, washed with water, dried and recrystallized with ethanol. 2-(4-(2-Chloroacetyl)piperazin-1-yl)-*N*-(2-(4-chlorophenyl)-4-oxoquinazolin-3(4*H*)-yl)acetamide (**V**) (0.01 mol) was reacted with different corresponding aniline (0.01 mol) with a few drops of glacial acetic acid in absolute ethanol was refluxed for 10 h (40 °C) to synthesize the title compounds (**1**–**17**). The reaction mixture was then cooled in ice cold water and the resultant precipitate was filtered, washed with water, dried and recrystallized with ethanol.

### Antimicrobial evaluation (in vitro)

The synthesized derivatives of quinazolinone were tested using ciprofloxacin (antibacterial) and fluconazole (antifungal) as reference drugs for their in vitro antimicrobial potential. The antimicrobial activity towards Gram-positive bacteria: *S. aureus* MTCC3160, *B. subtilis* MTCC441, Gram-negative bacterium: *E. coli* MTCC443 and fungal species: *C. albicans* MTCC227 and *A. niger* MTCC281 was determined by tube dilution method [33]. To give a concentration of 100 µg/ml, the standard and test samples were dissolved in DMF. Dilutions of test and standard compounds were prepared in double strength nutrient broth I.P. (bacteria) or Sabouraud dextrose broth I.P. (fungi) [34].

### Anticancer evaluation (in vitro)

It has been determined the activity of synthesized compounds and control drugs against human colon (HCT116) and cancer cell lines of the mouse monocyte macrophage leukaemic (RAW 264.7). In RPMI 1640 (Sigma) the selected cancer cell strains were developed, supplemented by 10% heat-inactivated bovine fetal serum (FBS) (PAA Laboratories) and 1% penicillin/streptomycin (PAA Laboratories). Cultures were kept at 37 °C in a humidified incubator in an environment of 5% CO_2_. Anticancer activity of synthesized compounds was assessed at distinct concentrations using MTT assay, as Mosmann (1983) explained but with minor alteration after 72 h of incubation. Using a spectrophotometer at 520 nm, assay sheets were read. Data produced were used to determine a dose–response curve from which the concentration of test compounds needed to kill 50% of the cell population (IC_50_) was determined [35].

### Molecular docking

#### Target identification

Kinase inhibitors are extremely efficient in the therapy of cancer, specifically targeting specific mutations that mainly drive tumorigenesis. They are categorized according to their capacity to catalyze ATP terminal phosphate transfer to substrates that usually contain serine, threonine or tyrosine residues [36]. Cycline-dependent kinases (CDKs) are a family of significant regulatory proteins that regulate different cell activity and are primarily engaged in the cell cycle and transcription. It is not surprising that many diseases, especially cancer, are common in their aberrant activities, given the fundamental biological functions played by CDKs. Different cell cycle proteins such as CDKs and cyclines induce development of the cell cycle as they are the main regulators of the cell cycle. Previous trials have shown that quinazoline derivatives therapy arrests cancer cells in the G2/M stage CDK activity enables the orderly transition between cell cycle stages. Cell cycle progression inhibition and apoptosis are the most prevalent causes of inhibition of cell growth [37]. The macrophage-like cell line RAW264.7 promotes replication of murine noroviruses as opposed to most other mouse-derived cell cultures and is commonly used for this purpose. In addition, RAW264.7 was discovered to be unique in research of a mouse model of serious respiratory disease for the propagation of the causative agent, pneumonia virus of mice and for the measurement of pro-inflammatory mediators associated with infection [38, 39].

#### Docking

To investigate the binding mode of synthesized quinazolinone compounds and standard drugs with selected PDB ID for cancer cell lines, molecular docking research was implemented i.e. human colorectal carcinoma and mouse leukaemic monocyte macrophage, was retrieved from protein data bank. GLIDE (Schrodinger 2018-1, maestro *v11.5*) acquired the docking score and targeted the binding site and formed the grid. The active site grid covered the major amino acids that interact with the receptor. Using their specific PDB ID: 5FGK for human colorectal carcinoma and PDB ID: 5JVY for mouse monocyte macrophage (Additional file 1), the protein’s 3-D structure was acquired from the protein database. The protein structure has been prepared using the protein preparation wizard in the Schrodinger maestro *v11.5*. A set of quinazolinone compounds have been selected as ligands for the docking studies and their structures have been drawn using the workspace of the maestro and converted into 3D form [40, 41].

## Results and discussion

### Chemistry

**Scheme 1:**
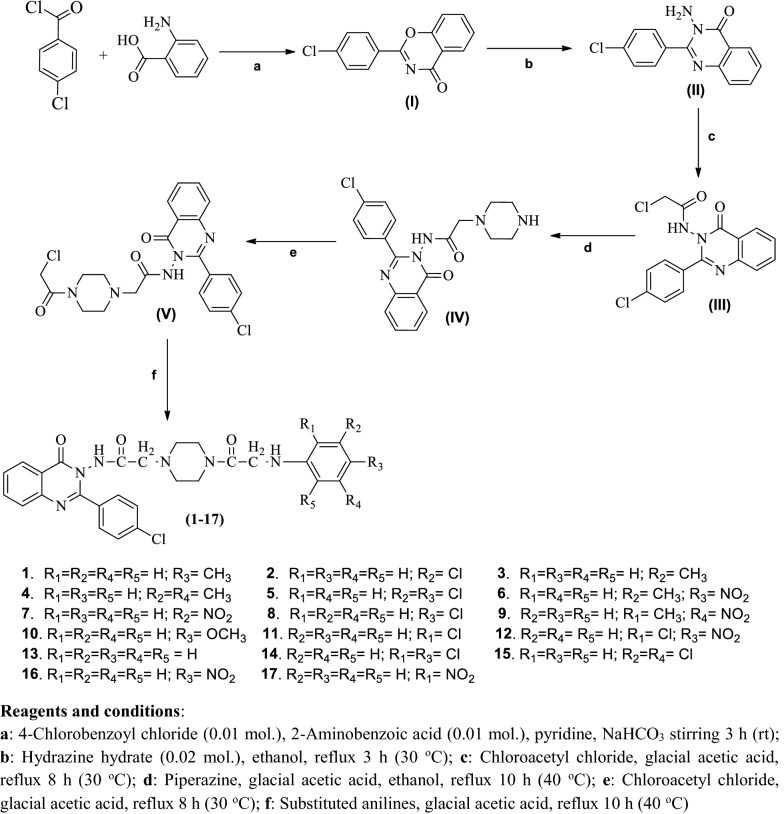
Synthesis of 2-(4-(2-chloroacetyl)piperazin-1-yl)-*N*-(2-(4-chlorophenyl)-4-oxoquinazolin-3(4*H*)-yl)acetamide derivatives **1**–**17**

A new series of quinazoline derivatives was synthesized using synthetic Scheme 1 (**1**–**17**) (Additional file 2). The physicochemical and spectral characteristics of the synthesized derivatives are presented in Tables 1 and 2, respectively. The molecular structures of the quinazoline compounds were confirmed by FT-IR, ^1^H-NMR, ^13^C–NMR, MS and elemental analysis. The IR spectrum of compound showed around 2928–3077 cm^−1^ and 1561–1592 cm^−1^ indicated the presence of C–H and C=C groups, respectively. The presence of C=O group in compounds displayed in the scale of 1665–1727 cm^−1^. The presence of an arylalkyl ether group (Ar-OCH_3_) in synthesized compounds is established by the existence of an IR absorption band around 1195 cm^−1^. The appearance of IR stretching 1591–1636 cm^−1^ in the spectral data of compounds (**1**–**17**) specified the existence of N=CH group. The appearance of IR stretching 1250–1253 cm^−1^ in the spectral data of synthesized compounds specified the existence of C–N group. The proton NMR displayed the multiplet signals between 6.03 and 8.05 δ ppm in the aromatic ring of the synthesized compounds. The compounds exhibited singlet at 8.91 δ ppm due to the existence of N–NH group. Compounds displayed singlet at 2.36–2.38 δ ppm due to the existence of –CH_3_ group. The compounds displayed singlet at 3.71δ ppm due to the existence of OCH_3_ of Ar-OCH_3_. The ^13^C–NMR spectra of aromatic ring exhibited the carbon atoms in the range of 170.9, 164.9, 164.6, 161.3, 151.9, 138.4, 135.8, 135.5, 133.5, 132.3, 129.5, 129.4, 128.0, 127.9, 127.6, 127.4, 126.9, 122.4, 121.7, 120.6, 118.3, 114.6, 58.7, 52.5, 52.4, 52.3, 46.8, 46.7 of the synthesized compounds. The elemental analysis was found within ± 0.4% of the theoretical results of compounds.

**Table 1:** The physicochemical properties of the synthesized compounds

Compound no.	M. formula	M. W.	M. P. (°C)	R_f_ value^a^	% yield
**1.**	C_29_H_29_ClN_6_O_3_	545.0	150–152	0.51	91.74
**2.**	C_28_H_26_Cl_2_N_6_O_3_	565.5	162–164	0.51	76.90
**3.**	C_29_H_29_ClN_6_O_3_	545.0	156–158	0.13	18.24
**4.**	C_30_H_31_ClN_6_O_3_	559.1	162–164	0.53	53.03
**5.**	C_28_H_25_Cl_3_N_6_O_3_	599.9	108–110	0.20	69.19
**6.**	C_29_H_28_ClN_7_O_5_	590.0	126–128	0.19	81.53
**7.**	C_28_H_26_ClN_7_O_5_	576.0	156–158	0.56	92.64
**8.**	C_28_H_26_Cl_2_N_6_O_3_	565.5	128–130	0.43	77.08
**9.**	C_29_H_28_ClN_7_O_5_	590.0	138–140	0.15	77.04
**10.**	C_29_H_29_ClN_6_O_4_	561.0	156–158	0.50	59.89
**11.**	C_28_H_26_Cl_2_N_6_O_3_	565.5	118–120	0.38	84.18
**12.**	C_28_H_25_Cl_2_N_7_O_5_	610.4	110–112	0.57	77.70
**13.**	C_28_H_27_ClN_6_O_3_	531.0	160–162	0.40	74.43
**14.**	C_28_H_25_Cl_3_N_6_O_3_	599.9	120–122	0.29	49.42
**15.**	C_28_H_25_Cl_3_N_6_O_3_	599.9	168–170	0.20	76.90
**16.**	C_28_H_26_ClN_7_O_5_	576.0	122–124	0.24	85.78
**17.**	C_28_H_26_ClN_7_O_5_	576.0	152–154	0.31	44.61

^a^TLC mobile phase: benzene

**Table 2:** Spectral characteristics of the synthesized compounds

Compd. no.	IUPAC nomenclature	IR (KBr pellets, cm^−1^)	^1^H-NMR and ^13^C–NMR (DMSO δ, ppm)	C, H, N analysis and MS
**1.**	*N*-(2-(4-Chlorophenyl)-4-oxoquinazolin-3(4H)-yl)-2-(4-(2-(p-tolyl-amino)acetyl)piperazin-1-yl)acetamide	IR: 1669 (C=O str., CONH_2_), 3214 (N–H str., 2° NH_2_), 1469 (N–N str.), 1250 (C–N str.), 3067 (C–H str., Ar), 1590 (C=C str., Ar), 1634 (C= N str., Ar), 2920 (C–H str., R-CO–CH_2_), 725 (C–Cl str., Ar–Cl), 2853 (C–H sym str., Ar-CH_3_)	^1^H-NMR: 7.38–8.05 (m, 12H, ArH), 8.19 (s, 1H, N–NH), 4.15 (d, 2H, CH_2_), 2.38 (s, 3H CH_3_), 3.32 (t, 4H, CH_2_ of piperazine), 2.63 (t, 4H, CH_2_ of piperazine); ^13^C–NMR: 170.2, 164.6, 164.2, 161.4, 151.7, 149.3, 135.6, 135.4, 133.1, 131.2, 129.3, 129.5, 128.8, 127.4, 127.2, 126.6, 122.5, 120.5, 117.5, 113.6, 111.7, 58.5, 53.5, 52.7, 52.5, 46.9, 46.5	Anal cal. C, 63.91; H, 5.36; N, 15.42; Found: C, 63.94; H, 5.32; N, 15.40; MS ES+ (ToF): *m/z* 546 [M^+^ + 1]
**2.**	*N*-(2-(4-Chlorophenyl)-4-oxoquinazolin-3(4H)-yl)-2-(4-(2-((3-chloro-phenyl)amino)acetyl)- piperazin-1-yl)acetamide	IR: 1668 (C=O str., CONH_2_), 3213 (N–H str., 2° NH_2_), 1468 (N–N str.), 1252 (C–N str.), 3067 (C–H str., Ar), 1591 (C=C str., Ar), 1632 (C= N str., Ar), 2957 (C–H str., R-CO–CH_2_), 1727 (C=O str., Ar-ketone), 726 (C–Cl str., Ar–Cl)	^1^H-NMR: 6.55–7.86 (m, 12H, ArH), 8.19 (s, 1H, N–NH), 4.15 (d, 2H, CH_2_), 3.33 (t, 4H, CH_2_ of piperazine), 2.64 (t, 4H, CH_2_ of piperazine); ^13^C–NMR: 170.0, 164.6, 164.3, 161.3, 151.0, 145.5, 135.5, 133.5, 129.9, 129.8, 129.6, 129.5, 128.7, 127.1, 127.2, 127.3, 126.2, 122.7, 122.4, 120.9, 114.6, 58.6, 53.4, 52.1, 52.0, 46.9, 46.7	Anal cal. C, 59.47; H, 4.63; N, 14.86; Found: C, 59.49; H, 4.67; N, 14.83; MS ES+ (ToF): *m/z* 566 [M^+^ + 1]
**3.**	*N*-(2-(4-Chlorophenyl)-4-oxoquinazolin-3(4H)-yl)-2-(4-(2-(m-tolyl-amino)acetyl)piperazin-1-yl)acetamide	IR: 1669 (C=O str., CONH_2_), 3214 (N–H str., 2° NH_2_), 1467 (N–N str.), 1272 (C–N str.), 3068 (C–H str., Ar), 1592 (C=C str., Ar), 1632 (C= N str., Ar), 2958 (C–H str., R-CO–CH_2_), 1726 (C=O str., Ar-ketone), 727 (C–Cl str., Ar–Cl), 2863 (C–H sym str., Ar-CH_3_)	^1^H-NMR: 7.00–7.74 (m, 11H, ArH), 8.19 (s, 1H, N–NH), 4.15 (d, 2H, CH_2_), 3.34 (t, 4H, CH_2_ of piperazine), 2.63 (t, 4H, CH_2_ of piperazine), 2.36 (s, 3H, CH_3_); ^13^C–NMR: 170.2, 164.7, 164.6, 161.0, 151.1, 136.7, 136.6, 135.3, 133.4, 129.6, 129.2, 128.6, 127.4, 127.2, 127.1, 127.0, 126.1, 122.0, 120.7, 116.5, 114.4, 114.0, 58.8, 53.6, 52.4, 52.3, 46.8, 46.7, 15.6	Anal cal. C, 63.91; H, 5.36; N, 15.42; Found: C, 63.95; H, 5.40; N, 15.48; MS ES+ (ToF): *m/z* 545 [M^+^ + 1]
**4.**	*N*-(2-(4-Chlorophenyl)-4-oxoquinazolin-3(4H)-yl)-2-(4-(2-((3,5-dimethylphenyl)amino)-acetyl)piperazin-1-yl)acetamide	IR: 1669 (C=O str., CONH_2_), 3213 (N–H str., 2° NH_2_), 1467 (N–N str.), 1253 (C–N str.), 3066 (C–H str., Ar), 1588 (C=C str., Ar), 1634 (C= N str., Ar), 2957 (C–H str., R-CO–CH_2_), 1726 (C=O str., Ar-ketone), 725 (C–Cl str., Ar–Cl), 2862 (C–H sym str., Ar-CH_3_)	^1^H-NMR: 7.55–7.86 (m, 11H, ArH), 8.19 (s, 1H, N–NH), 4.16 (d, 2H, CH_2_), 3.30 (t, 4H, CH_2_ of piperazine), 2.65 (t, 4H, CH_2_ of piperazine), 2.37 (s, 3H, CH_3_); ^13^C–NMR: 170.3, 164.9, 164.7, 161.2, 151.4, 143.4, 135.4, 133.3, 131.5, 129.7, 129.3, 128.3, 127.3, 127.0, 126.8, 126.6, 126.4, 126.3, 122.2, 120.4, 113.5, 58.9, 53.8, 52.1, 52.0, 46.9, 46.4, 24.7, 15.6	Anal cal. C, 64.45; H, 5.59; N, 15.03; Found: C, 64.47; H, 5.56; N, 15.05; MS ES+ (ToF): *m/z* 560 [M^+^ + 1]
**5.**	*N*-(2-(4-Chlorophenyl)-4-oxoquinazolin-3(4H)-yl)-2-(4-(2-((3,4-dichlorophenyl)-amino)acetyl)piperazin-1-yl)acetamide	IR: 1665 (C=O str., CONH_2_), 3216 (N–H str., 2° NH_2_), 1463 (N–N str.), 1252 (C–N str.), 3056 (C–H str., Ar), 1591 (C=C str., Ar), 1611 (C= N str., Ar), 2923 (C–H str., R-CO–CH_2_), 1709 (C=O str., Ar-ketone), 724 (C–Cl str., Ar–Cl)	^1^H-NMR: 6.72–7.86 (m, 11H, ArH), 8.19 (s, 1H, N–NH), 4.15 (d, 2H, CH_2_), 3.32 (t, 4H, CH_2_ of piperazine), 2.64 (t, 4H, CH_2_ of piperazine); ^13^C–NMR: 170.5, 164.9, 164.7, 161.5, 151.3, 145.6, 135.0, 134.3, 133.2, 129.8, 129.4, 129.2, 128.5, 127.5, 127.1, 126.6, 123.2, 122.5, 120.7, 118.9, 113.1, 58.8, 52.8, 52.4, 46.3, 46.8	Anal cal. C, 56.06; H, 4.20; N, 14.01; Found: C, 56.06; H, 4.20; N, 14.01; MS ES+ (ToF): *m/z* 600 [M^+^ + 1]
**6.**	*N*-(2-(4-Chlorophenyl)-4-oxoquinazolin-3(4H)-yl)-2-(4-(2-((3-methyl-4-nitrophenyl)-amino)acetyl)piperazin-1-yl)acetamide	IR: 1669 (C=O str., CONH_2_), 3211 (N–H str., 2° NH_2_), 1474 (N–N str.), 1251 (C–N str.), 1590 (C=C str., Ar), 1634 (C= N str., Ar), 2928 (C–H str., R-CO–CH_2_), 1729 (C=O str., Ar-ketone), 725 (C–Cl str., Ar–Cl), 2862 (C–H sym str., Ar-CH_3_), 1559 (NO_2_ asym str.)	^1^H-NMR: 6.45–8.05 (m, 11H, ArH), 8.19 (s, 1H, N–NH), 4.15 (d, 2H, CH_2_), 3.33 (t, 4H, CH_2_ of piperazine), 2.63 (t, 4H, CH_2_ of piperazine), 3.54 (s, 3H, OCH_3_); ^13^C–NMR: 170.6, 164.6, 164.4, 161.1, 152.7, 151.5, 136.8, 135.2, 133.4, 129.6, 129.4, 128.3, 127.4, 127.3, 126.3, 125.2, 122.7, 120.9, 118.6, 114.5, 58.5, 53.5, 52.5, 46.9, 46.0, 52.5, 14.3	Anal cal. C, 57.47; H, 4.66; N, 16.18; Found: C, 57.46; H, 4.68; N, 16.19; MS ES+ (ToF): *m/z* 591 [M^+^ + 1]
**7.**	*N*-(2-(4-Chlorophenyl)-4-oxoquinazolin-3(4H)-yl)-2-(4-(2-((3-nitrophenyl)amino)-acetyl)piperazin-1-yl)acetamide	IR: 1668 (C=O str., CONH_2_), 3213 (N–H str., 2° NH_2_), 1467 (N–N str.), 1253 (C–N str.), 3066 (C–H str., Ar), 1588 (C=C str., Ar), 1632 (C= N str., Ar), 2958 (C–H str., R-CO–CH_2_), 1727 (C=O str., Ar-ketone), 725 (C–Cl str., Ar–Cl), 1558 (NO_2_ asym str.)	^1^H-NMR: 6.73–7.85 (m, 12H, ArH), 8.19 (s, 1H, N–NH), 4.16(d, 2H, CH_2_), 3.32 (t, 4H, CH_2_ of piperazine), 2.63 (t, 4H, CH_2_ of piperazine); ^13^C–NMR: 170.5, 164.5, 164.2, 161.0, 151.6, 149.4, 148.3, 135.5, 133.5, 130.8, 129.3, 129.0, 128.5, 127.6, 127.2, 127.1, 126.3, 122.8, 120.7, 119.4, 107.6, 109.7, 58.3, 53.0, 52.6, 52.3, 46.8, 46.2	Anal cal. C, 58.39; H, 4.55; N, 17.02; Found: C, 58.42; H, 4.56; N, 17.01; MS ES+ (ToF): *m/z* 577 [M^+^ + 1]
**8.**	*N*-(2-(4-Chlorophenyl)-4-oxoquinazolin-3(4H)-yl)-2-(4-(2-((4-chlorophenyl)amino)- acetyl)-piperazin-1-yl)acetamide	IR: 1667 (C=O str., CONH_2_), 3214 (N–H str., 2° NH_2_), 1469 (N–N str.), 1250 (C–N str.), 3067 (C–H str., Ar), 1592 (C=C str., Ar), 1630 (C= N str., Ar), 2958 (C–H str., R-CO–CH_2_), 1720 (C=O str., Ar-ketone), 725 (C–Cl str., Ar–Cl), 2862 (C–H sym str., Ar-CH_3_)	^1^H-NMR: 6.09–8.03 (m, 12H, ArH), 8.19 (s, 1H, N–NH), 4.15 (d, 2H, CH_2_), 3.31 (t, 4H, CH_2_ of piperazine), 2.66 (t, 4H, CH_2_ of piperazine); ^13^C–NMR: 170.2, 164.6, 164.2, 161.4, 151.7, 149.3, 135.6, 135.4, 131.2, 133.1, 129.5, 129.3, 128.8, 127.4, 127.2, 126.6, 122.5, 120.5, 117.5, 113.6, 111.7, 58.5, 52.7, 52.5, 53.5, 46.9, 46.5	Anal cal. C, 59.47; H, 4.63; N, 14.86; Found: C, 59.43; H, 4.68; N, 14.88; MS ES+ (ToF): *m/z* 565 [M^+^ + 1]
**9.**	*N*-(2-(4-Chlorophenyl)-4-oxoquinazolin-3(4H)-yl)-2-(4-(2-((2-methyl-5-nitrophenyl) amino)-acetyl)piperazin-1-yl)acetamide	IR: 1668 (C=O str., CONH_2_), 3213 (N–H str., 2° NH_2_), 1468 (N–N str.), 3068 (C–H str., Ar), 1589 (C=C str., Ar), 1631 (C= N str., Ar), 2957 (C–H str., R-CO–CH_2_), 1726 (C=O str., Ar-ketone), 725 (C–Cl str., Ar–Cl), 1252 (C–N str., piperazine), 1558 (NO_2_ asym str.)	^1^H-NMR: 7.16–7.74 (m, 11H, ArH), 8.19 (s, 1H, N–NH), 4.15(d, 2H, CH_2_), 3.33 (t, 4H, CH_2_ of piperazine), 2.63 (t, 4H, CH_2_ of piperazine), 2.38 (s, 3H, CH_3_); ^13^C–NMR: 170.5, 164.4, 164.0, 161.2, 151.6, 147.5, 146.5, 135.7, 133.0, 132.6, 130.9, 129.6, 129.5, 128.5, 127.6, 127.5, 127.1, 126.9, 122.4, 120.3, 109.7, 107.5, 58.7, 53.4, 52.9, 52.6, 46.8, 46.6, 15.7	Anal cal. C, 59.03; H, 4.78; N, 16.62; Found: C, 59.01; H, 4.74; N, 16.60; MS ES+ (ToF): *m/z* 591 [M^+^ + 1]
**10.**	*N*-(2-(4-Chlorophenyl)-4-oxoquinazolin-3(4H)-yl)-2-(4-(2-((4-methoxyphenyl) amino)acetyl)-piperazin-1-yl)-acetamide	IR: 1670 (C=O str., CONH_2_), 3215 (N–H str., 2° NH_2_), 1470 (N–N str.), 1250 (C–N str.), 3065 (C–H str., Ar), 1591 (C=C str., Ar), 1635 (C= N str., Ar), 1776 (C=O str., Ar-ketone), 725 (C–Cl str., Ar–Cl), 2892 (C–H str., R-CO–CH_2_), 1195 (C–O–C str., Ar–O–CH_3_)	^1^H-NMR: 7.55–7.88 (m, 12H, ArH), 8.19 (s, 1H, N–NH), 4.15(d, 2H, CH_2_), 3.33 (t, 4H, CH_2_ of piperazine), 2.64 (t, 4H, CH_2_ of piperazine), 3.71 (s, 3H, OCH_3_); ^13^C–NMR: 170.5, 164.3, 161.2, 151.5, 149.4, 139.7, 135.9, 133.4, 129.4, 129.3, 128.7, 127.8, 127.7, 127.6, 126.6, 122.7, 120.8, 115.3, 115.2, 114.7, 114.6, 58.9, 55.4, 53.6, 52.7, 52.5, 46.7, 46.6	Anal cal. C, 62.08; H, 5.21; N, 14.98; Found: C, 62.09; H, 5.25; N, 14.99; MS ES+ (ToF): *m/z* 562 [M^+^ + 1]
**11.**	*N*-(2-(4-Chlorophenyl)-4-oxoquinazolin-3(4H)-yl)-2-(4-(2-((2-chlorophenyl)amino)-acetyl)piperazin-1-yl)acetamide	IR: 3177 (N–H str., 2° NH_2_), 1500 (N–N str.), 1247 (C–N str.), 3096 (C–H str., Ar), 1598 (C=C str., Ar), 1634 (C= N str., Ar), 2958 (C–H str., R-CO–CH_2_), 1725 (C=O str., Ar-ketone), 722 (C–Cl str., Ar–Cl)	^1^H-NMR: 7.22–7.71 (m, 11H, ArH), 8.19 (s, 1H, N–NH), 4.15(d, 2H, CH_2_), 3.31 (t, 4H, CH_2_ of piperazine), 2.65 (t, 4H, CH_2_ of piperazine); ^13^C–NMR: 170.5, 164.8, 164.2, 161.0, 151.3, 150.1, 138.4, 135.5, 133.2, 129.3, 129.1, 128.4, 127.7, 127.4, 127.3, 126.9, 122.6, 124.7, 123.5, 120.3, 120.1, 115.6, 58.6, 52.9, 52.7, 52.5, 46.6, 46.2	Anal cal. C, 55.09; H, 4.13; N, 16.06; Found: C, 55.06; H, 4.17; N, 16.09; MS ES+ (ToF): *m/z* 566 [M^+^ + 1]
**12.**	2-(4-(2-((2-Chloro-4-nitrophenyl)amino)acetyl)piperazin-1-yl)-*N*-(2-(4-chloro-phenyl)-4-oxoquinazolin-3(4H)-yl)acetamide	IR: 1669 (C=O str., CONH_2_), 3214 (N–H str., 2° NH_2_), 1479 (N–N str.), 1251 (C–N str.), 3071 (C–H str., Ar), 1591 (C=C str., Ar), 1636 (C= N str., Ar.), 2958 (C–H str., R-CO–CH_2_), 1726 (C=O str., Ar-ketone), 724 (C–Cl str., Ar–Cl)	^1^H-NMR: 6.60–7.85 (m, 11H, ArH), 8.19 (s, 1H, N–NH), 4.16(d, 2H, CH_2_), 3.31 (t, 4H, CH_2_ of piperazine), 2.65 (t, 4H, CH_2_ of piperazine); ^13^C–NMR: 170.6, 164.5, 164.3, 161.4, 151.5, 149.7, 146.7, 135.6, 133.4, 130.7, 129.6, 129.3, 128.5, 127.9, 127.5, 127.1, 126.7, 122.7, 121.2, 120.3, 117.5, 108.9, 58.8, 53.6, 52.9, 52.8, 46.7, 46.4	Anal cal. C, 55.09; H, 4.13; N, 16.06; Found: C, 55.07; H, 4.17; N, 16.03; MS ES+ (ToF): *m/z* 611 [M^+^ + 1]
**13.**	*N*-(2-(4-Chlorophenyl)-4-oxoquinazolin-3(4H)-yl)-2-(4-(2-(phenylamino)acetyl) piperazin-1-yl)acetamide	IR: 1668 (C=O str., CONH_2_), 3214 (N–H str., 2° NH_2_), 1468 (N–N str.), 1251 (C–N str.), 3067 (C–H str., Ar), 1590 (C=C str., Ar), 1631 (C= N str., Ar), 2928 (C–H str., R-CO–CH_2_), 1725 (C=O str., Ar-ketone), 725 (C–Cl str., Ar–Cl)	^1^H-NMR: 6.50–7.73 (m, 13H, ArH), 8.19 (s, 1H, N–NH), 4.19 (d, 2H, CH_2_), 3.31 (t, 4H, CH_2_ of piperazine), 2.65 (t, 4H, CH_2_ of piperazine); ^13^C–NMR: 170.9, 164.7, 164.0, 161.5, 151.7, 147.8, 135.9, 133.1, 129.9, 129.6, 129.4, 129.2, 128.3, 127.6, 127.5, 127.2, 126.4, 122.9, 120.6, 117.5, 113.6, 113.3, 58.9, 53.4, 52.8, 52.4, 46.9, 46.6	Anal cal. C, 63.33; H, 5.13; N, 15.83; Found: C, 63.38; H, 5.17; N, 15.81; MS ES+ (ToF): *m/z* 632 [M^+^ + 1]
**14.**	*N*-(2-(4-Chlorophenyl)-4-oxoquinazolin-3(4H)-yl)-2-(4-(2-((2,4-dichlorophenyl)amino)-acetyl)piperazin-1-yl)acetamide	IR: 1668 (C=O str., CONH_2_), 3213 (N–H str., 2° NH_2_), 1467 (N–N str.), 1274 (C–N str.), 3066 (C–H str., Ar), 1592 (C=C str., Ar), 1632 (C= N str., Ar), 2928 (C–H str., R-CO–CH_2_), 1726 (C=O str., Ar-ketone), 726 (C–Cl str., Ar–Cl)	^1^H-NMR: 6.35–7.69 (m, 11H, ArH), 8.19 (s, 1H, N–NH), 4.15 (d, 2H, CH_2_), 3.32 (t, 4H, CH_2_ of piperazine), 2.65 (t, 4H, CH_2_ of piperazine); ^13^C–NMR: 170.6, 164.8, 164.1, 161.8, 151.4, 147.4, 135.8, 134.3, 133.0, 131.4, 129.2, 129.0, 128.7, 127.5, 127.4, 127.3, 126.7, 122.6, 121.7, 120.7, 115.5, 113.2, 58.5, 53.5, 52.5, 52.2, 46.8, 46.5	Anal cal. C, 56.06; H, 4.20; N, 14.01; Found: C, 56.08; H, 4.22; N, 14.04; MS ES+ (ToF): *m/z* 601 [M^+^ + 1]
**15.**	*N*-(2-(4-Chlorophenyl)-4-oxoquinazolin-3(4H)-yl)-2-(4-(2-((3,5-dichlorophenyl)amino)-acetyl)piperazin-1-yl)acetamide	IR: 1668 (C=O str., CONH_2_), 3214 (N–H str., 2° NH_2_), 1468 (N–N str.), 1251 (C–N str.), 3072 (C–H str., Ar), 1591 (C=C str., Ar), 1632 (C=N str., Ar), 2956 (C–H str., R-CO–CH_2_), 1725 (C=O str., Ar-ketone), 725 (C–Cl str., Ar–Cl)	^1^H-NMR: 7.55–7.86 (m, 12H, ArH), 8.19 (s, 1H, N–NH), 4.14 (d, 2H, CH_2_), 3.31 (t, 4H, CH_2_ of piperazine), 2.64 (t, 4H, CH_2_ of piperazine); ^13^C–NMR: 170.4, 164.6, 164.0, 161.5, 151.1, 143.7, 135.5, 133.2, 129.8, 129.2, 129.0, 128.4, 127.7, 127.6, 127.5, 127.3, 126.9, 122.3, 122.5, 120.8, 118.4, 114.5, 58.3, 52.7, 52.4, 52.3, 46.9, 46.7	Anal cal. C, 59.47; H, 4.63; N, 14.86; Found: C, 59.49; H, 4.61; N, 14.87; MS ES+ (ToF): *m/z* 601 [M^+^ + 1]
**16.**	*N*-(2-(4-Chlorophenyl)-4-oxoquinazolin-3(4H)-yl)-2-(4-(2-((4-nitrophenyl)amino)acetyl)-piperazin-1-yl)acetamide	IR: 1669 (C=O str., CONH_2_), 3217 (N–H str., 2° NH_2_), 1469 (N–N str.), 1254 (C–N str.), 3077 (C–H str., Ar), 1561 (C=C str., Ar), 1629 (C= N str., Ar), 2958 (C–H str., R-CO–CH_2_), 1726 (C=O str., Ar-ketone), 725 (C–Cl str., Ar–Cl), 1561 (NO_2_ asym str.)	^1^H-NMR: 6.43–7.76 (m, 12H, ArH), 8.19 (s, 1H, N–NH), 4.14 (d, 2H, CH_2_), 3.31 (t, 4H, CH_2_ of piperazine), 2.63 (t, 4H, CH_2_ of piperazine); ^13^C–NMR: 170.7, 164.8, 164.3, 161.6, 153.8, 151.5, 136.6, 135.6, 133.0, 129.3, 129.1, 128.2, 127.8, 127.5, 127.4, 126.8, 122.1, 121.7, 120.9, 114.6, 114.5, 58.4, 53.2, 52.4, 52.2, 46.9, 46.6	Anal cal. C, 58.39; H, 4.55; N, 17.02; Found: C, 58.40; H, 4.57; N, 17.04; MS ES+ (ToF): *m/z* 577 [M^+^ + 1]
**17.**	*N*-(2-(4-Chlorophenyl)-4-oxoquinazolin-3(4H)-yl)-2-(4-(2-((2-nitrophenyl)amino)acetyl)-piperazin-1-yl)acetamide	IR: 1668 (C=O str., CONH_2_), 3213 (N–H str., 2° NH_2_), 1467 (N–N str.), 1253 (C–N str.), 3067 (C–H str., Ar), 1590 (C=C str., Ar), 1632 (C= N str., Ar), 2957 (C–H str., R-CO–CH_2_), 1726 (C=O str., Ar-ketone), 726 (C–Cl str., Ar–Cl), 1559 (NO_2_ asym str.)	^1^H-NMR: 6.63–7.76 (m, 12H, ArH), 8.19 (s, 1H, N–NH), 4.17 (d, 2H, CH_2_), 3.32 (t, 4H, CH_2_ of piperazine), 2.51 (t, 4H, CH_2_ of piperazine); ^13^C–NMR: 170.9, 164.9, 164.6, 161.3, 151.9, 138.4, 135.8, 135.5, 133.5, 132.3, 129.5, 129.4, 128.0, 127.9, 127.6, 127.4, 126.9, 122.4, 121.7, 120.6, 118.3, 114.6, 58.7, 52.5, 52.4, 52.3, 46.8, 46.7	Anal cal. C, 58.39; H, 4.55; N, 17.02; Found: C, 58.37; H, 4.51; N, 17.01; MS ES+ (ToF): *m/z* 577 [M^+^ + 1]

### Antimicrobial screening results

The synthesized compounds were tested by tube dilution technique against Gram positive and Gram negative bacterial and fungal strains for their in vitro antimicrobial activity and the MIC values of control drugs and synthetic compounds are shown in Table 3. Antimicrobial screening results indicated that compound **3** (MIC_bs_ = 4.81 µM) emerged as most effective antibacterial agent toward *B. subtilis.* Compounds **11** and **12** (MIC_sa_ = 2.56 µM**)** displayed the promising activity toward *S. aureus.* Compound **8** (MIC_ec_ = 5.54 µM) emerged as most active candidate against Gram-negative bacterium *E. coli* and comparable to standard drug ciprofloxacin. In the case of antifungal activity, compound **3** (MIC_ca_ = 4.81 and MIC_an_ = 2.40 µM) was found to be the most potent antifungal agent against *C. albicans* and *A. niger* and had better antifungal activity than standard drug fluconazole (MIC_ca,an_ = 5.09 µM). Results of antimicrobial activity indicated that the synthesized compounds possessed a higher antifungal activity than antibacterial activity. As far as antibacterial activity is concerned, the synthesized compounds were more active against Gram negative bacterium (*E. coli*) than Gram positive bacteria (*S. aureus* and *B. subtilis*).

**Table 3:** Antimicrobial screening results of the synthesized compounds

Compound no.	Minimum inhibitory concentration (MIC = µM)				
	Bacterial strains			Fungal strains	
	*B. subtilis*	*S. aureus*	*E. coli*	*C. albicans*	*A. niger*
**1.**	91.74	11.47	22.94	11.47	91.74
**2.**	22.12	88.50	11.06	22.12	11.06
**3.**	4.81	19.23	9.62	4.81	2.40
**4.**	22.36	11.18	89.45	11.18	89.45
**5.**	83.47	10.43	83.47	10.43	20.87
**6.**	21.19	21.19	21.19	10.59	5.30
**7.**	21.70	10.85	86.81	10.85	86.81
**8.**	22.16	11.08	5.54	88.65	11.08
**9.**	10.59	21.19	84.75	10.59	21.19
**10.**	11.14	22.28	89.13	89.13	11.14
**11.**	20.49	2.56	20.49	10.25	81.97
**12.**	81.97	2.56	81.97	20.49	5.12
**13.**	11.77	23.54	11.77	23.54	94.16
**14.**	10.43	20.87	83.47	20.87	83.47
**15.**	83.33	83.33	10.42	20.83	20.83
**16.**	86.81	10.85	86.81	10.85	21.70
**17.**	21.70	86.81	21.70	10.85	21.70
Std.	4.71^a^	4.71^a^	4.71^a^	5.09^b^	5.09^b^
DMSO	NA	NA	NA	NA	NA
Broth control	NG	NG	NG	NG	NG

The samples were incubated at 37 ± 1 °C for 24 h (bacteria), at 25 ± 1 °C for 7d (*A. niger*) and at 37 ± 1 °C for 48 h (*C. albicans*), respectively and the results were recorded in terms of MIC (the lowest concentration of test substance which inhibited the growth of microorganisms)

Std.: ^a^Ciprofloxacin, ^b^Fluconazole; *NA* no activity, *NG* no growth

### Anticancer screening results

The synthesized compounds were evaluated for their in vitro anticancer activity against human colorectal cancer (HCT116) and mouse monocyte macrophage (RAW 264.7) cancer cell lines using MTT assay. Compound **5** (IC_50_ = 0.82 and 12.39 µM against RAW 264.7 and HCT116, respectively) was found to be the most active one and compared to the 5-fluorouracil and Tomudex (used as standard drugs). The anticancer screening (IC_50_ = µM) results are shown in Table 4.

**Table 4:** Anticancer screening results of the synthesized compounds

Compound no.	Anticancer screening (IC_50_ = µM)	
	Cancer cell lines	
	HCT116	RAW 264.7
**1**.	119.26	1.30
**2.**	93.73	3.36
**3.**	91.78	1.95
**4.**	20.27	8.53
**5.**	12.39	0.82
**6.**	27.12	5.08
**7.**	24.31	1.20
**8.**	99.27	14.36
**9.**	103.38	15.93
**10.**	52.28	4.63
**11.**	114.67	16.38
**12.**	81.91	3.06
**13.**	94.14	17.70
**14.**	87.23	26.67
**15.**	84.89	17.45
**16.**	30.09	9.55
**17.**	14.64	1.74
Raltitrexed (Tomudex)	9.05	2.81
5-Fluorouracil	4.60	0.60

### Molecular docking results

**Fig. 3:**
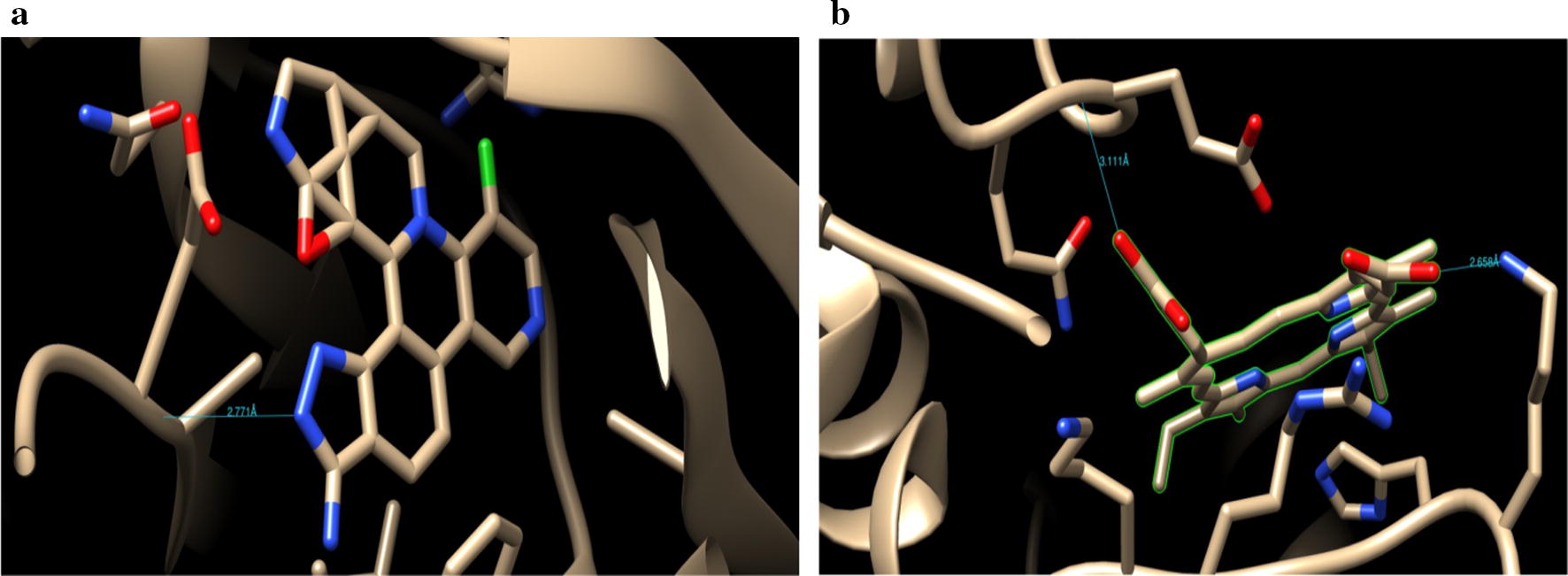
Binding surface and ligand interaction diagram of native ligand 5XG (**a**) and COH (**b**) with their respective protein

**Fig. 4:**
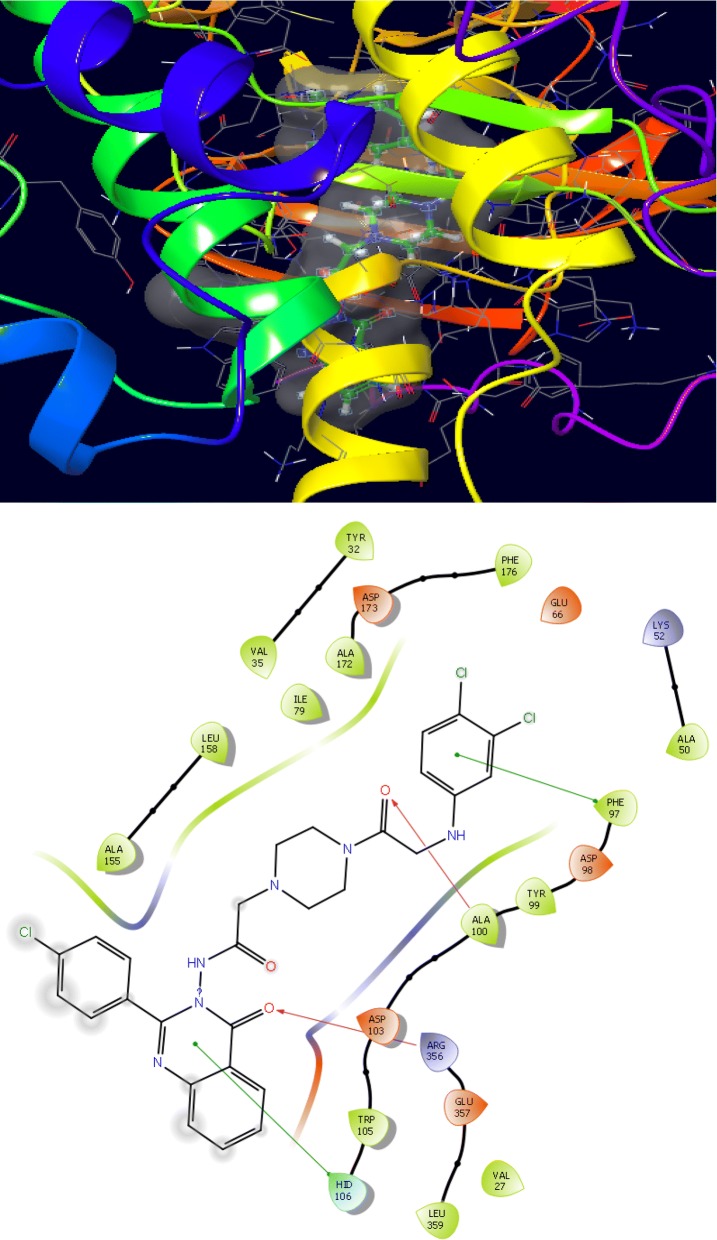
Binding surface and ligand interaction diagram of compound **5**

**Fig. 5:**
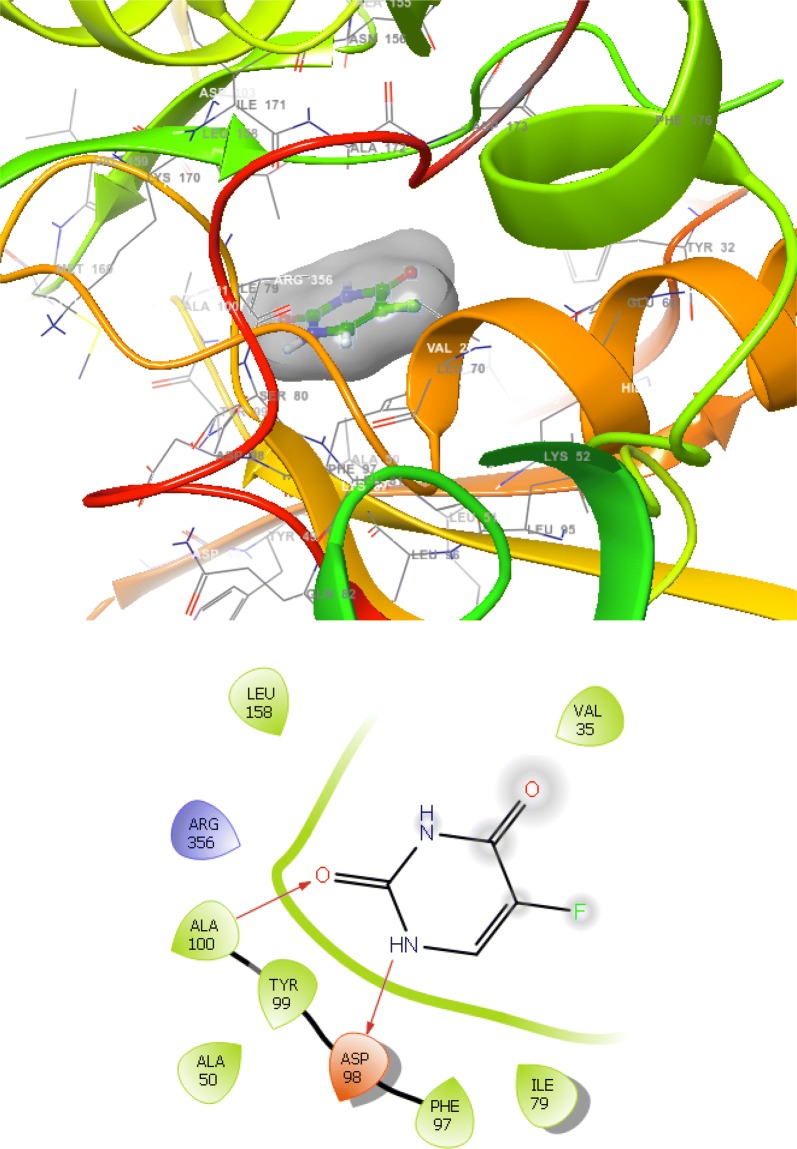
Binding surface and ligand interaction diagram of 5-fluorouracil

**Fig. 6:**
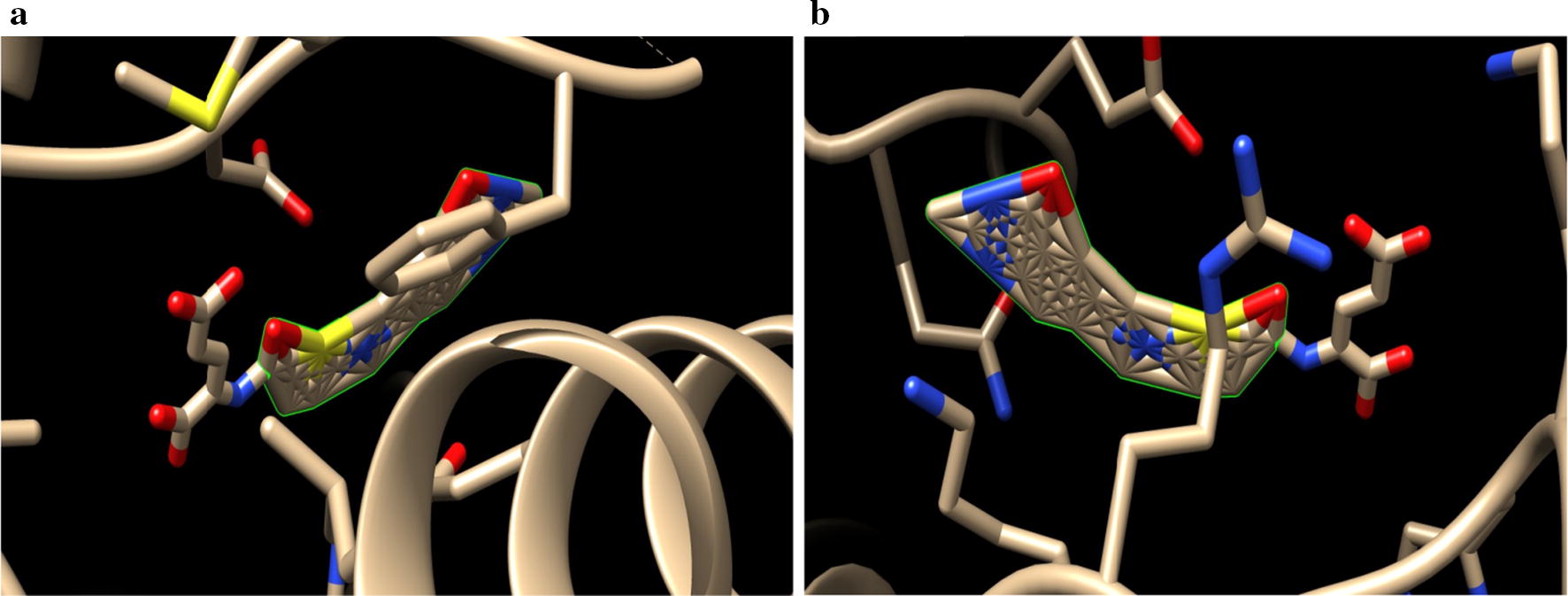
Binding surface and ligand interaction diagram of Raltitrexed (Tomudex) with 5FGK (**a**) and 5JVY (**b**) proteins

**Fig. 7:**
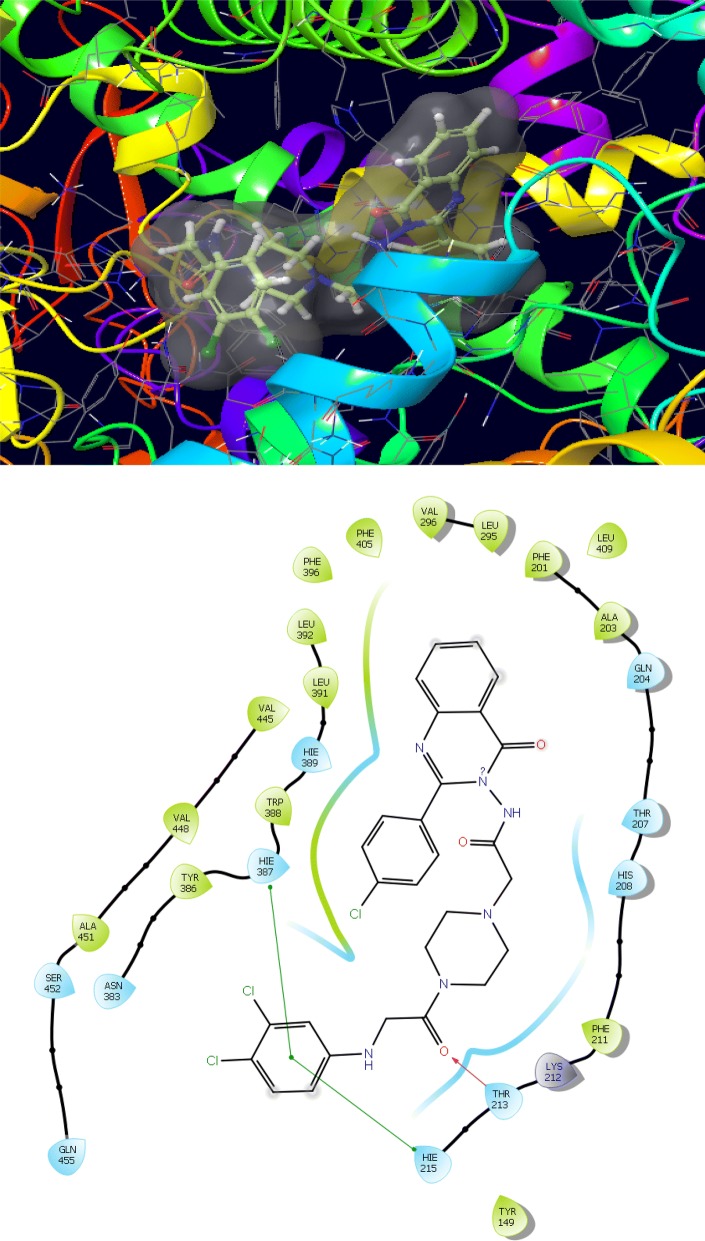
Binding surface and ligand interaction diagram of compound **5**

**Fig. 8:**
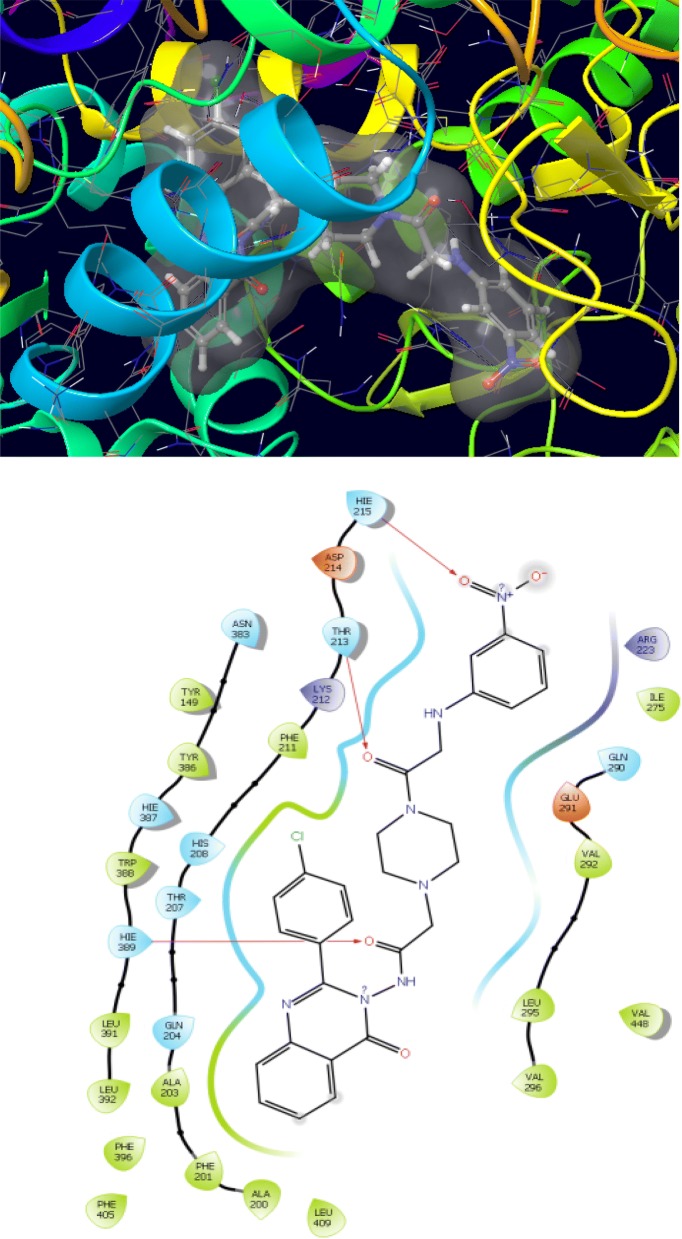
Binding surface and ligand interaction diagram of compound **7**

**Fig. 9:**
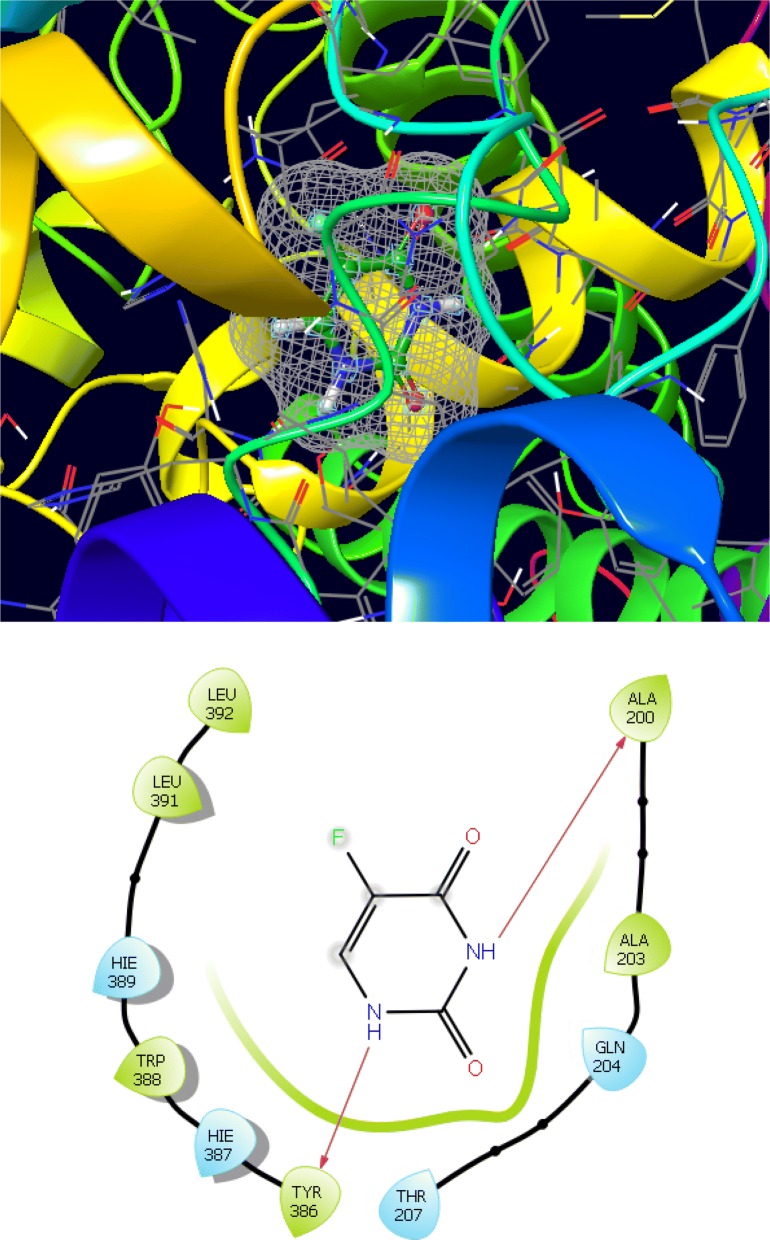
Binding surface and ligand interaction diagram of 5-fluorouracil

The synthesized derivatives of quinazolinone showed excellent docking performance and the chosen proteins were discovered to communicate with significant amino acids. Molecular docking was performed to analyze the binding mode of active compounds **5** and **7** for the cancer cell lines of human colorectal carcinoma (HCT116) and mouse leukaemic monocyte macrophage (RAW 264.7). The compound **5** and standard drugs (5-fluorouracil and tomudex) were docked in the active site of the cycline-dependent kinase cdk8 (PDB ID: 5FGK) co-crystallized acuity 5XG ligand and in the active site of the S121P murine COX-2 mutant (PDB ID: 5JVY) co-crystallized acuity ligand COH were also docked. The binding mode of native ligand 5XG have docked score   (− 8.72) and COH ligand have docked score (− 8.93) showed good interaction with crucial amino acids residues with their respective proteins (Fig. 3). The binding mode of compound **5** (Fig. 4) (using PDB ID: 5FGK for HCT116), scored docked score (− 8.011) with moderate anticancer potency (12.39 μM) and formed H-bond with crucial amino acids and compared to the docked score of 5-fluorouracil (Fig. 5) have lowest docked score (− 5.753) with better anticancer activity (4.6 μM) and tomudex (− 10.86) have good docked score with better anticancer potency (9.05 μM) (Fig. 6a). The binding mode of active compound **5** (using PDB ID: 5JVY for mouse leukaemic monocyte macrophage) have docked score (− 11.054) with better anticancer activity (0.82 μM) which also developed H-bond with crucial amino acid (Fig. 7) and compound **7** have docked score (− 11.284) with better anticancer potency (1.20 μM) (Fig. 8) and comparable to 5-fluorouracil have lowest docked score (− 4.122) with better anticancer potency (0.60 μM) (Fig. 9) and also compared to the tomudex (− 10.83) have better docked score with better anticancer potency (2.81 μM) (Fig. 6b). The docking results of synthesized compounds (**5** and **7**), native ligands and standard drug (tomudex) which showed good to better docking score with their respective proteins. Docking results and interacting residues are shown in Tables 5 and 6.

**Table 5:** Molecular docking results and interacting residues of compound **5** and standard drugs

Compound no.	Docking score	Glide energy (kcal/mol)	Interacting residues
**5**	− 8.011	− 64.796	Ala155, Leu158, Ile79, Ala172, Asp173, Phe176, VaL35, Tyr32, Glu66, Lys52, Ala50, Phe97, Asp98, Tyr99, Ala100, Asp103, Trp105, Hid106, Arg356, Glu357, Leu359, Val27
5XG	− 8.72	− 49.49	Ile79, Ala172, Asp173, Arg356, Phe97, Ala 100, Val159, Glu101, Gly33
Raltitrexed (Tomudex)	− 10.86	− 54.30	Met174, Asp173, Phe176, Glu66A, Lys52, Leu70, Ile79
5-Fluorouracil	− 5.753	− 21.673	Leu158, Arg356, Ala100, Tyr99, Asp98, Phe97, Ile79, Ala50, Val35

**Table 6:** Molecular docking results and interacting residues of compounds **5, 7** and standard drugs

Compound no.	Docking score	Glide energy (kcal/mol)	Interacting residues
**5**	− 11.054	− 68.766	Gln455, Ser452, Ala451, Val448, Val445, Asn383, Tyr386, Hie387, Trp388, Hie389, Leu391, Leu392, Phe396, Phe405, Val296, Leu295, Phe201, Ala203, Gln204, Thr207, His208, Phe211, Lys212, Thr213, Hie215, Tyr149, Leu409
**7**	− 11.284	− 71.663	Phe405, Phe396, Leu392, Leu391, Hie389, Trp388, Hie387, Tyr386, Tyr149, Asn383, Leu409, Ala200, Phe201, Ala203, Gln204, Thr207, His208, Phe211, Lys212, Thr213, Asp214, Hie215, Val296, Leu295, Val292, Glu291, Gln290, Ile275, Arg223, Val448
COH	− 8.93	− 54.81	Glu291, Lys216, Arg223, Lys212, Gln290, His 215
Raltitrexed (Tomudex)	− 10.83	− 58.73	Glu291, Arg223, Lys212, Gln290, Thr238, Glu 209
5-Fluorouracil	− 4.122	− 26.585	Leu392, Leu391, Hie389, Trp388, Hie387, Tyr386, Thr207, Gln204, Ala203, Ala200

The docking findings therefore indicate that the synthesized compounds may be of excellent importance in effective chemotherapy. The selected database of protein i.e. (PDB ID: 5FGK) for human colorectal carcinoma and (PDB ID: 5JVY) mouse monocyte macrophage may be the target protein of derivatives of quinazolinone for their anticancer activity (Additional file 3).

## Structure activity relationship studies (SAR)

**Fig. 10:**
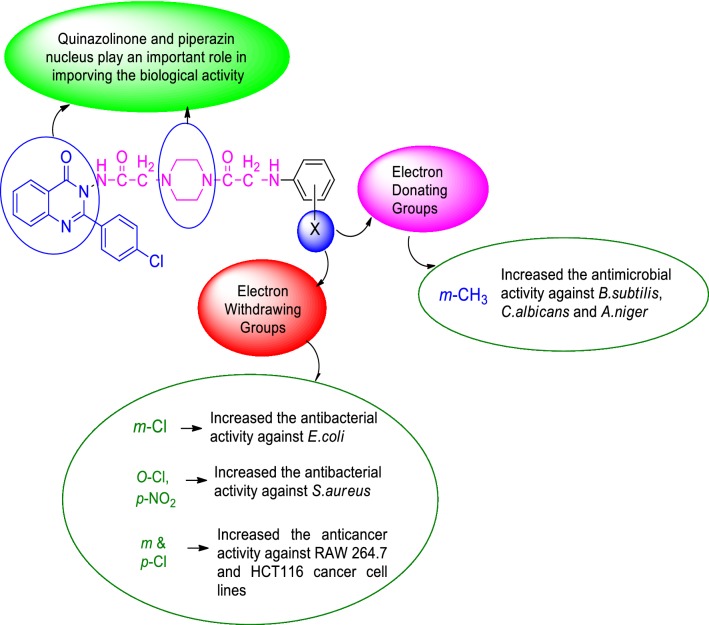
Structure activity relationship study of the synthesized compounds

The SAR of synthesized compounds can be summarized (Fig. 10) as follow:

- Results of antimicrobial activity indicated that substitution of phenyl amino ring attached to the chlorophenyl quinazolinone piperazine-acetamide nucleus with electron donating methyl group enhanced the antimicrobial activity against *B. subtilis, C. albicans* and *A. niger.*
- In the case of antibacterial activity towards *S. aureus* and *E. coli,* electron withdrawing groups i.e. chloro (Cl) and chloro with nitro (NO_2_) substitution on the phenylamino ring attached to the chlorophenyl quinazolinone piperazine-acetamide nucleus increased the antibacterial activity.
- Results of anticancer activity revealed that the presence of electron withdrawing groups i.e. dichloro on the phenylamino ring attached to the chlorophenyl quinazolinone piperazine acetamide nucleus increased the anticancer activity against both cancer cell lines (RAW 264.7 and HCT116).

## Conclusion

In the present study, the synthesized quinazolinone derivatives i.e. compound **3** showed promising antimicrobial activity due to the presence of electron releasing group at *meta*–position of the substituted benzylidene nucleus and comparable to the control drugs. In case of anticancer activity indicated that compound **5** (*meta/para*-Cl) and compound **7** (*meta-*NO_2_) displayed moderate anticancer activity towards human colorectal carcinoma and mouse leukaemic monocyte macrophage cancer cell lines due to the presence of EWG on the substituted benzylidene nucleus. Molecular docking analysis demonstrated that compounds **5** and **7** showed the better docked score with better potency and comparable to the standard drug and native ligands of the proteins. The findings of the docking are compatible with the assays of anticancer. Docking information stay in excellent correlation with the outcomes of anticancer activity and these molecules may be used as a lead in the design of new anticancer agents.

## Supplementary information

**Additional file 1.** Web link for PDB ID: 5FGK and 5JVY proteins.

**Additional file 2.** Synthetic scheme with chemical structures.

**Additional file 3.** Docking results of active compounds.

## Abbreviations

ALK: activin-like kinase
EGFR: epidermal growth factor receptor
PBMC: peripheral blood mononuclear cells
VEGFR: vascular endothelial growth factor
ATP: adinosine triphosphate
CDK: cyclin-dependent kinase
NO: nitric oxide
RNA: ribonucleic acid
NMR: nuclear magnetic resonance
IR: infrared
MS: mass spectrum
CHN: carbon hydrogen nitrogen
TLC: thin-layer chromatography
Str: starching
DMSO: dimethyl sulfoxide
rt: room temperature
FBS: fetal bovine serum
MIC: minimum inhibitory concentration
MTCC: Microbial Type Culture Collection
*E. coli*: *Escherichia coli*
*C. albicans*: *Candida albicans*
*S. aureus*: *Staphylococcus aureus*
*B. subtilis*: *Bacillus subtilis*
*A. niger*: *Aspergillus niger*
MTT: 3-(4,5-dimethylthiazol-2-yl)-2,5-diphenyltetrazolium bromide
HCT116: human colorectal carcinoma116
RAW 264.7: murine macrophaga 264.7
5-Fu: 5-fluorouracil
PDB: protein data bank
XP: extra precision
3D: 3 dimensional
