# Supplementary material for: Synthesis, molecular docking and biological potentials of new 2-(4-(2-chloroacetyl) piperazin-1-yl)-N-(2-(4-chlorophenyl)-4-oxoquinazolin-3(4H)-yl)acetamide derivatives
Source: BMC Chem. 2019 Sep 5;13(1):113. doi: 10.1186/s13065-019-0629-0 (PMC6727350; doi:10.1186/s13065-019-0629-0)
Supplement: Supplementary file 1 — Additional file 1. Web link for PDB ID: 5FGK and 5JVY proteins. [file 13065_2019_629_MOESM1_ESM.pdf]

Additional File 1

Web link for PDB ID: 5FGK

https://www.rcsb.org/structure/5FGK

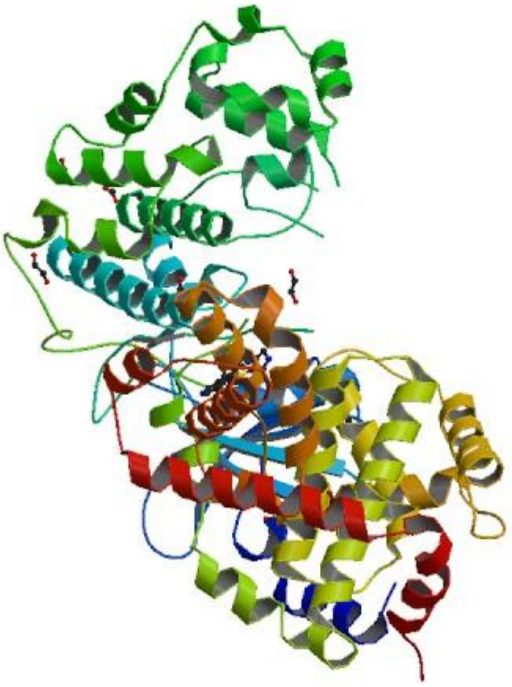

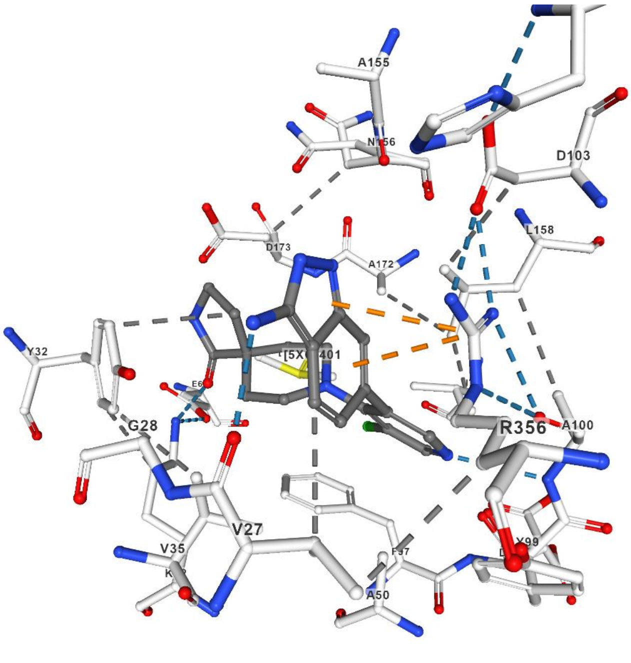

Protein structure with 5XG Ligand

Web link for PDB ID: 5JVY

https://www.rcsb.org/structure/5JVY

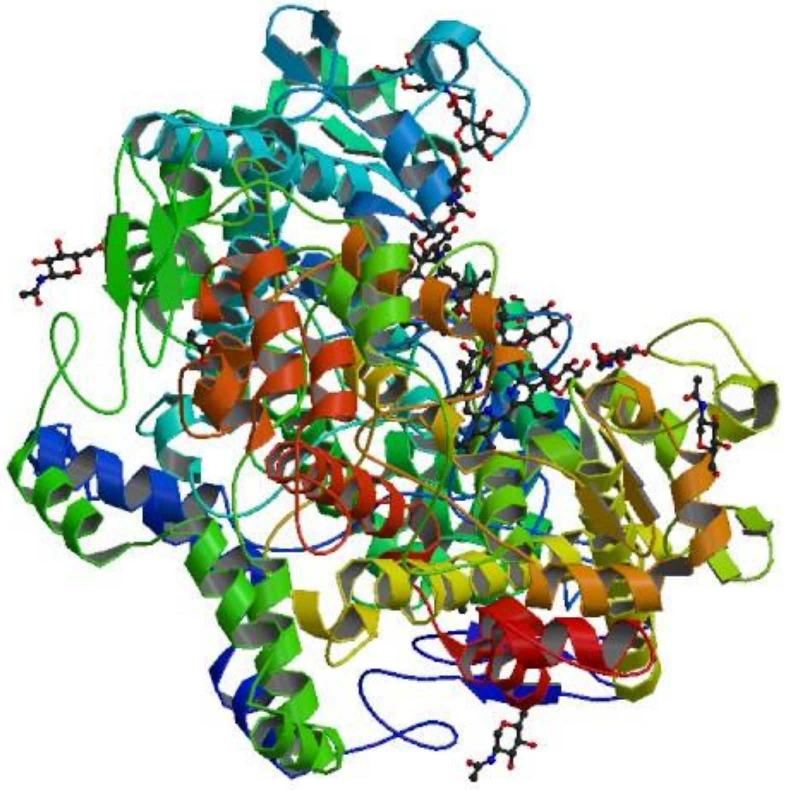

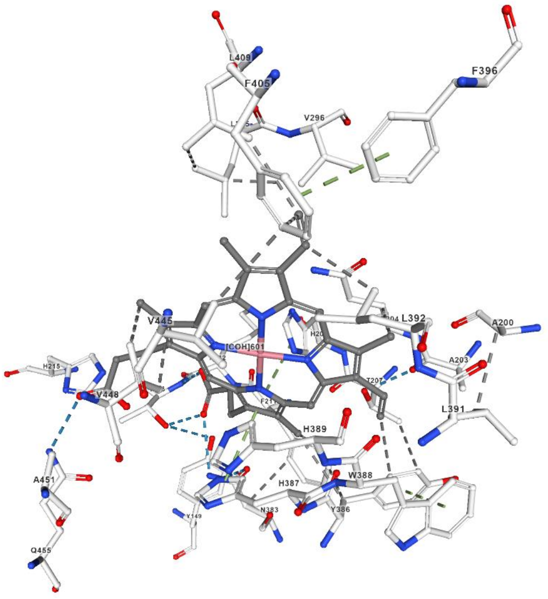

Protein structure with COH Ligand
