# Supplementary material for: Synthesis, molecular docking and biological potentials of new 2-(4-(2-chloroacetyl) piperazin-1-yl)-N-(2-(4-chlorophenyl)-4-oxoquinazolin-3(4H)-yl)acetamide derivatives
Source: BMC Chem. 2019 Sep 5;13(1):113. doi: 10.1186/s13065-019-0629-0 (PMC6727350; doi:10.1186/s13065-019-0629-0)
Supplement: Supplementary file 2 — Additional file 2. Synthetic scheme with chemical structures. [file 13065_2019_629_MOESM2_ESM.pdf]

Additional File 2

Synthetic scheme

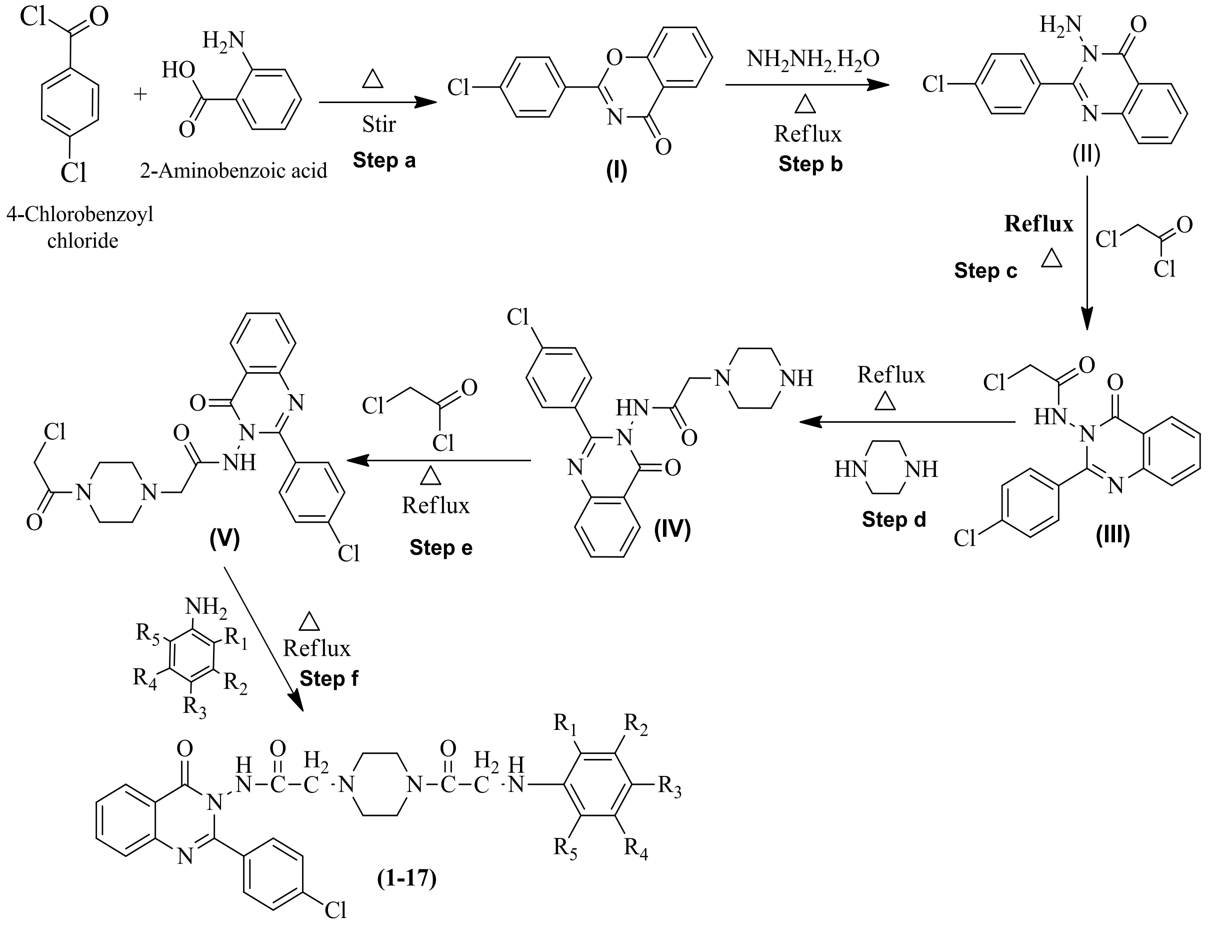

1. R_1=R_2=R_4=R_5= H; R_3= CH_3

2. R_1=R_3=R_4=R_5= H; R_2= Cl

3. R_1=R_3=R_4=R_5= H; R_2= CH_3

4. R_1=R_3=R_5= H; R_2=R_4= CH_3

5. R_1=R_4=R_5= H; R_2=R_3= Cl

6. R_1=R_4=R_5= H; R_2= CH_3; R_3= NO_2

7. R_1=R_3=R_4=R_5= H; R_2= NO_2

8. R_1=R_2=R_4=R_5= H; R_3= Cl

9. R_2=R_3=R_5= H; R_1= CH_3; R_4= NO_2

10. R_1=R_2=R_4=R_5= H; R_3= OCH_3

11. R_2=R_3=R_4=R_5= H; R_1= Cl

12. R_2=R_4= R_5= H; R_1= Cl; R_3= NO_2

13. R_1=R_2=R_3=R_4=R_5 = H

14. R_2=R_4=R_5= H; R_1=R_3= Cl

15. R_1=R_3=R_5= H; R_2=R_4= Cl

16. R_1=R_2=R_4=R_5= H; R_3= NO_2

17. R_2=R_3=R_4=R_5= H; R_1= NO_2

Compound	Chemical structure
1	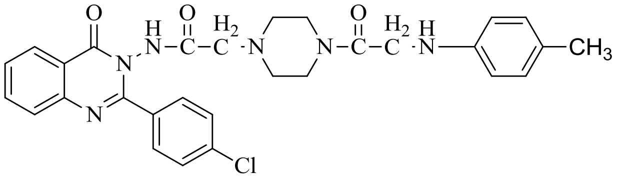 N-(2-(4-Chlorophenyl)-4-oxoquinazolin-3(4H)-yl)-2-(4-(2-(p-tolyl-amino)acetyl)piperazin-1-yl)acetamide

**2**	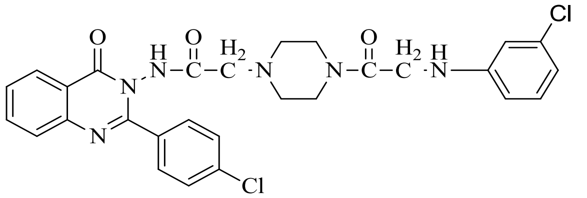 N-(2-(4-Chlorophenyl)-4-oxoquinazolin-3(4H)-yl)-2-(4-(2-((3-chloro-phenyl)amino)acetyl)- piperazin-1-yl)acetamide
**3**	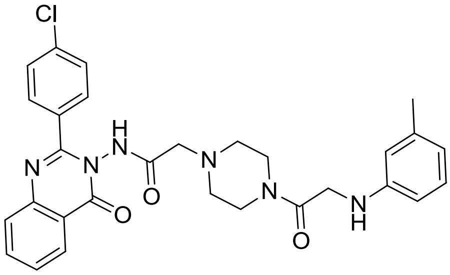 N-(2-(4-Chlorophenyl)-4-oxoquinazolin-3(4H)-yl)-2-(4-(2-(m-tolyl-amino)acetyl)piperazin-1-yl)acetamide
**4**	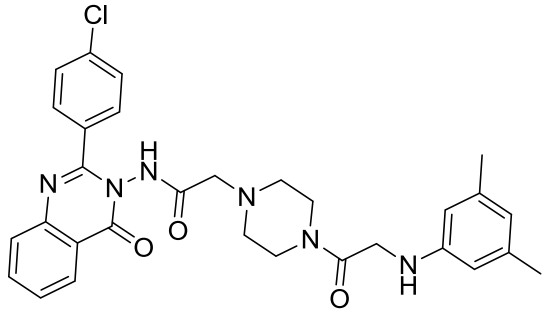 N-(2-(4-Chlorophenyl)-4-oxoquinazolin-3(4H)-yl)-2-(4-(2-((3,5-dimethylphenyl)amino)-acetyl)piperazin-1-yl)acetamide
**5**	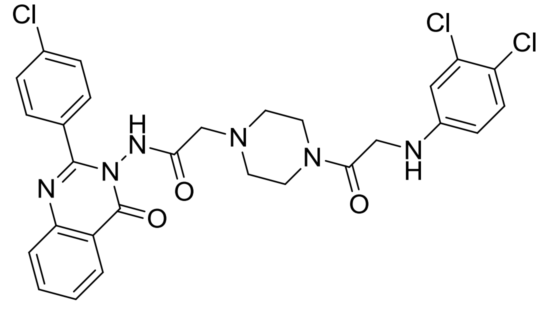 N-(2-(4-Chlorophenyl)-4-oxoquinazolin-3(4H)-yl)-2-(4-(2-((3,4-dichlorophenyl)-amino)acetyl)piperazin-1-yl)acetamide
**6**	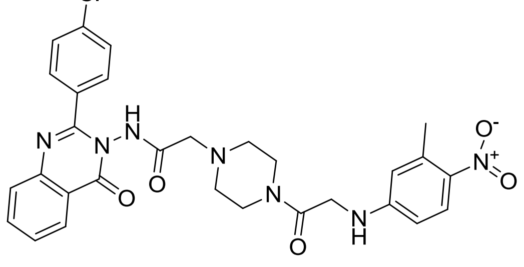 *N*-(2-(4-chlorophenyl)-4-oxoquinazolin-3(4*H*)-yl)-2-(4-(2-((3-methyl-4-nitrophenyl)amino)acetyl) piperazin-1-yl)acetamide

**7**	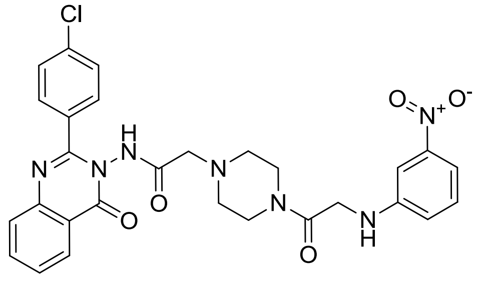 N-(2-(4-Chlorophenyl)-4-oxoquinazolin-3(4H)-yl)-2-(4-(2-((3-nitrophenyl) amino)-acetyl)piperazin-1-yl)acetamide
**8**	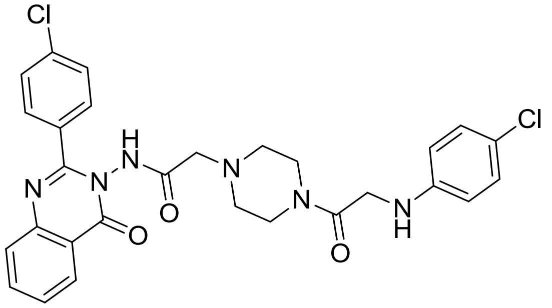 N-(2-(4-Chlorophenyl)-4-oxoquinazolin-3(4H)-yl)-2-(4-(2-((4-chlorophenyl) amino)- acetyl)-piperazin-1-yl)acetamide
**9**	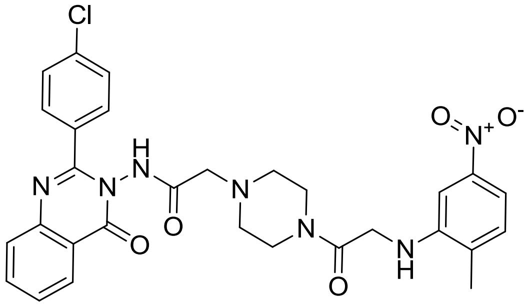 N-(2-(4-Chlorophenyl)-4-oxoquinazolin-3(4H)-yl)-2-(4-(2-((2-methyl-5-nitrophenyl) amino)-acetyl)piperazin-1-yl)acetamide
**10**	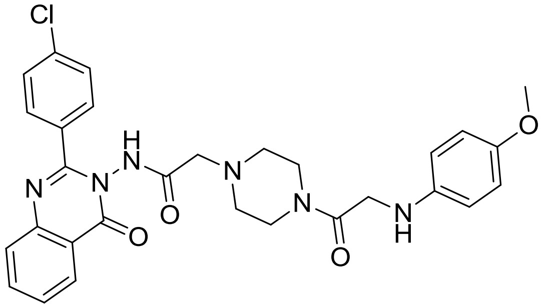 N-(2-(4-Chlorophenyl)-4-oxoquinazolin-3(4H)-yl)-2-(4-(2-((4-methoxyphenyl) amino)acetyl)-piperazin-1-yl)-acetamide

**11**	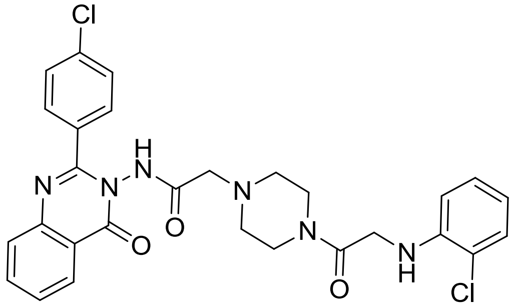<br>N-(2-(4-Chlorophenyl)-4-oxoquinazolin-3(4H)-yl)-2-(4-(2-((2-chlorophenyl)<br>amino)-acetyl)piperazin-1-yl)acetamide
**12**	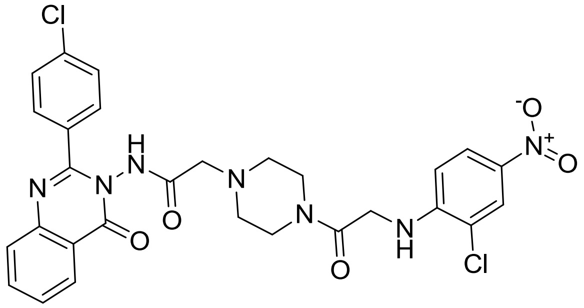<br>2-(4-(2-((2-Chloro-4-nitrophenyl)amino)acetyl)piperazin-1-yl)<br>-N-(2-(4-chloro-phenyl)-4-oxoquinazolin-3(4H)-yl)acetamide
**13**	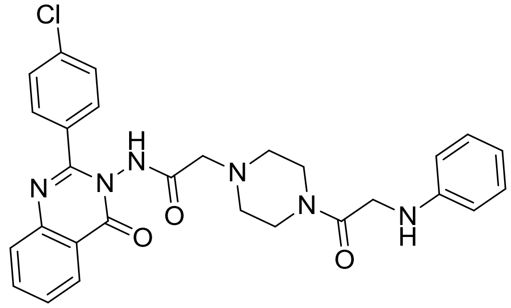<br>N-(2-(4-Chlorophenyl)-4-oxoquinazolin-3(4H)-yl)-2-(4-(2-(phenylamino)acetyl)<br>piperazin-1-yl)acetamide
**14**	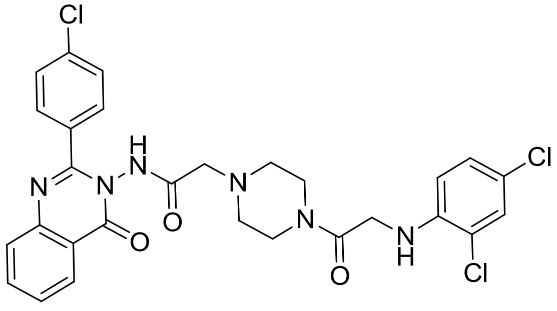<br>N-(2-(4-Chlorophenyl)-4-oxoquinazolin-3(4H)-yl)-2-(4-(2-((2,4-dichlorophenyl)amino)-<br>acetyl)piperazin-1-yl)acetamide

**15**	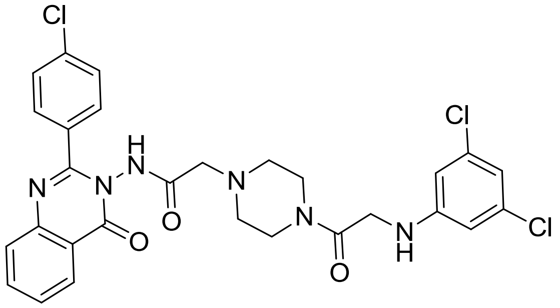<br>N-(2-(4-Chlorophenyl)-4-oxoquinazolin-3(4H)-yl)-2-(4-(2-((3,5-dichlorophenyl) amino)-acetyl)piperazin-1-yl)acetamide
**16**	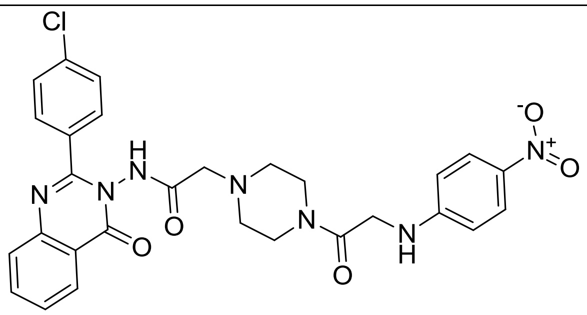<br>N-(2-(4-Chlorophenyl)-4-oxoquinazolin-3(4H)-yl)-2-(4-(2-((4-nitrophenyl) amino)acetyl)-piperazin-1-yl)acetamide
**17**	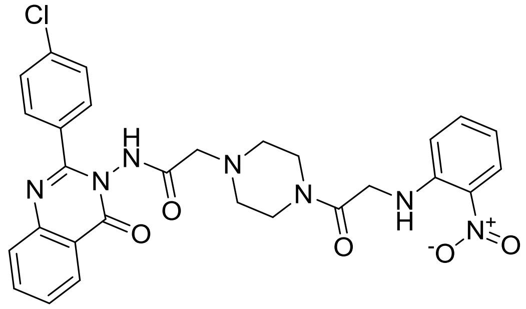<br>N-(2-(4-Chlorophenyl)-4-oxoquinazolin-3(4H)-yl)-2-(4-(2-((2-nitrophenyl) amino)acetyl)-piperazin-1-yl)acetamide
