# Supplementary material for: Synthesis, molecular docking and biological potentials of new 2-(4-(2-chloroacetyl) piperazin-1-yl)-N-(2-(4-chlorophenyl)-4-oxoquinazolin-3(4H)-yl)acetamide derivatives
Source: BMC Chem. 2019 Sep 5;13(1):113. doi: 10.1186/s13065-019-0629-0 (PMC6727350; doi:10.1186/s13065-019-0629-0)
Supplement: Supplementary file 3 — Additional file 3. Docking results of active compounds. [file 13065_2019_629_MOESM3_ESM.pdf]

Additional File 3

Molecular docking results PDB ID: 5FGK			
**Comp.**	**Docking Score**	**Glide energy (kcal/mol)**	**Interacting residues**
**5**	-8.011	-64.796	Ala155, Leu158, Ile79, Ala172, Asp173, Phe176, VaL35, Tyr32, Glu66, Lys52, Ala50, Phe97, Asp98, Tyr99, Ala100, Asp103, Trp105, Hid106, Arg356, Glu357, Leu359, Val27
**5XG**	-8.72	-49.49	Ile79, Ala172, Asp173, Arg356, Phe 97, Ala 100, Val159, Glu101, Gly33
**Raltitrexed (Tomudex)**	-10.86	-54.30	Met174, Asp173, Phe176, Glu66A, Lys52, Leu70, Ile79
**5-Fluorouracil**	-5.753	-21.673	Leu158, Arg356, Ala100, Tyr99, Asp98, Phe97, Ile79, Ala50, Val35

Pictorial presentation of the active compounds in 3D and 2D view

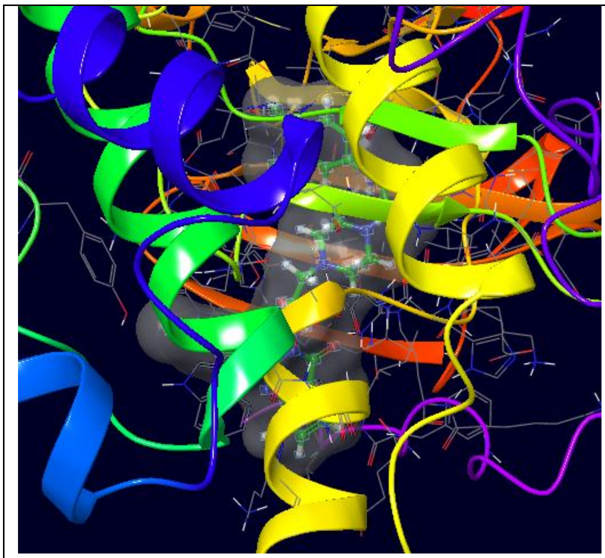

Pictorial presentation

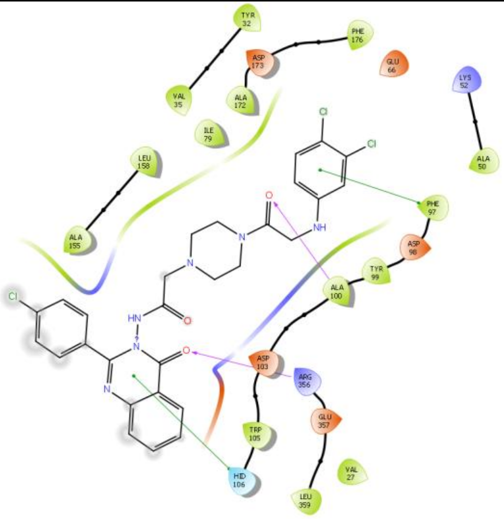

Ligand interaction diagram

Compound 5

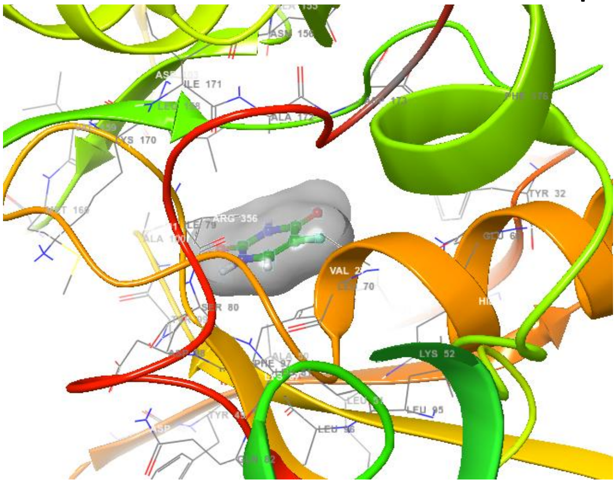

Pictorial presentation

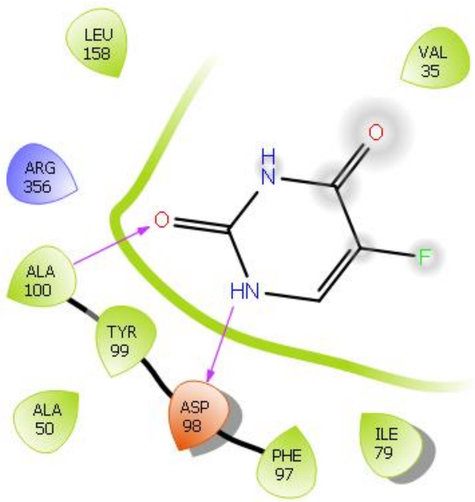

Ligand interaction diagram

5-Fluorouracil

Molecular docking results PDB ID: 5JVY			
**Comp.**	**Docking Score**	**Glide energy (kcal/mol)**	**Interacting residues**
**5**	-11.054	-68.766	Gln455, Ser452, Ala451, Val448, Val445, Asn383, Tyr386, Hie387, Trp388, Hie389, Leu391, Leu392, Phe396, Phe405, Val296, Leu295, Phe201, Ala203, Gln204, Thr207, His208, Phe211, Lys212, Thr213, Hie215, Tyr149, Leu409
**7**	-11.284	-71.663	Phe405, Phe396, Leu392, Leu391, Hie389, Trp388, Hie387, Tyr386, Tyr149, Asn383, Leu409, Ala200, Phe201, Ala203, Gln204, Thr207, His208, Phe211, Lys212, Thr213, Asp214, Hie215, Val296, Leu295, Val292, Glu291, Gln290, Ile275, Arg223, Val448
**COH**	-8.93	-54.81	Glu291, Lys216, Arg223, Lys212, Gln290, His 215
**Raltitrexed (Tomudex)**	-10.83	-58.73	Glu291, Arg223, Lys212, Gln290, Thr238, Glu 209
**5-Fluorouracil**	-4.122	-26.585	Leu392, Leu391, Hie389, Trp388, Hie387, Tyr386, Thr207, Gln204, Ala203, Ala200

Pictorial presentation of the active compounds in 3D and 2D view

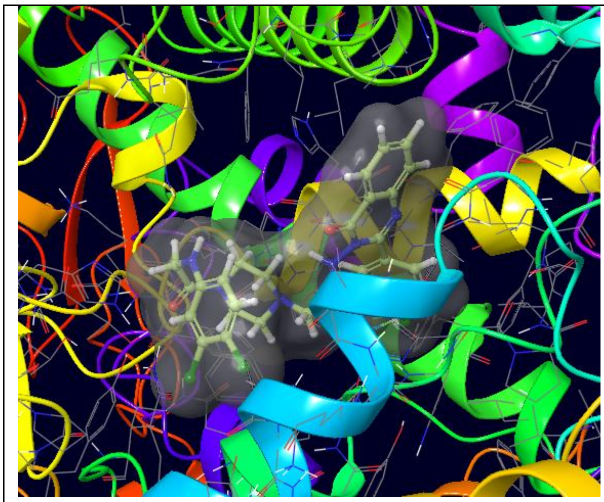

Pictorial presentation

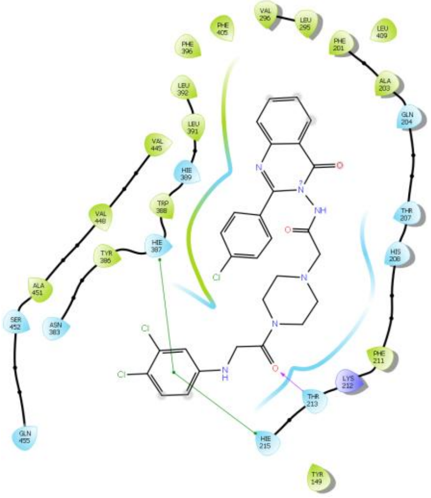

Ligand interaction diagram

Compound 5

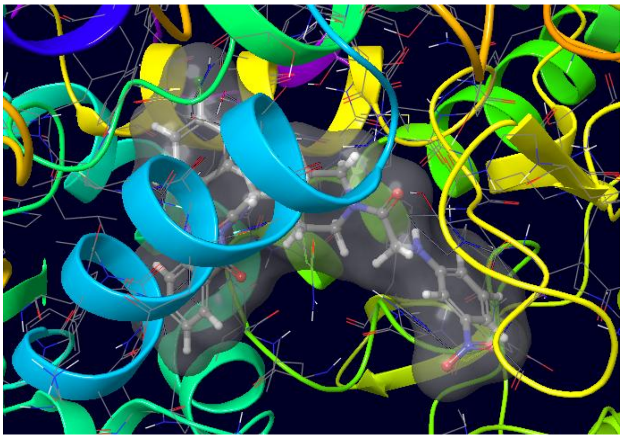

Pictorial presentation

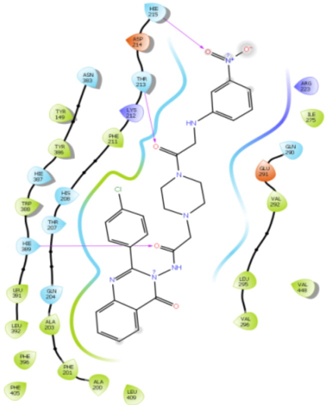

Ligand interaction diagram

Compound 7

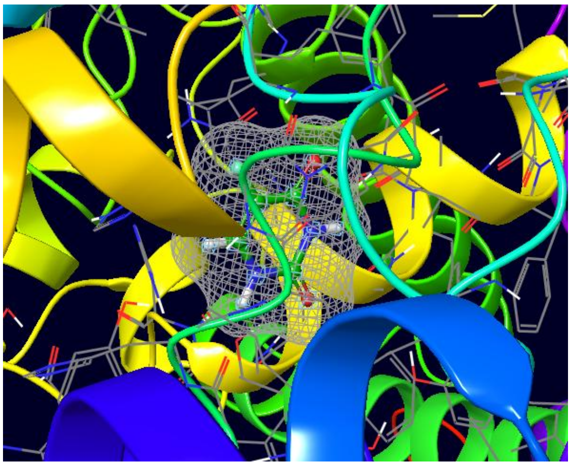

Pictorial presentation

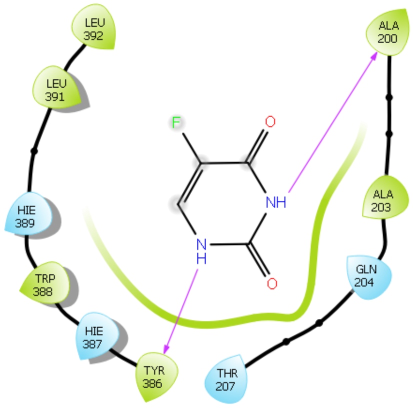

Ligand interaction diagram

5-Fluorouracil
